# Infection of 5xFAD mice with a mouse‐adapted SARS‐CoV‐2 does not alter Alzheimer's disease neuropathology yet induces widespread changes in gene expression across diverse cell types

**DOI:** 10.1002/alz.71394

**Published:** 2026-04-24

**Authors:** Susana Furman, Latifa Zayou, Kate Inman Tsourmas, Dominic Ibarra Javonillo, Gema M. Olivarria, Yuting Cheng, Collin Pachow, Kellie Fernandez, Lucas Le, Robert A. Edwards, Dequina A. Nicholas, Gabriela Pacheco Sanchez, Ralph S. Baric, Kim N. Green, Thomas E. Lane

**Affiliations:** ^1^ Department of Neurobiology and Behavior University of California Irvine California USA; ^2^ Department of Molecular Biology and Biochemistry School of Biological Sciences University of California Irvine California USA; ^3^ Department of Pathology & Laboratory Medicine School of Medicine University of California Irvine California USA; ^4^ Department of Epidemiology and Department of Microbiology &Immunology School of Medicine University of North Carolina Chapel Hill North Carolina USA; ^5^ Center for Virus Research University of California Irvine California USA

**Keywords:** Alzheimer's disease neuropathology, mouse‐adapted SARS‐CoV‐2, neuroinflammation, spatial transcriptomics, viral infection

## Abstract

**INTRODUCTION:**

The COVID‐19 pandemic underscored the impact of severe acute respiratory syndrome coronavirus 2 (SARS‐CoV‐2) infection on worsening the severity of Alzheimer's disease (AD) neuropathology and disease progression.

**METHODS:**

Aged 5xFAD and wild‐type (WT) mice were infected with a mouse‐adapted SARS‐CoV‐2 (MA10), and extensive characterization of molecular and cellular changes within the brains was carried out.

**RESULTS:**

MA10 infection induced acute viral pneumonia. Viral RNA was undetectable within the brains of infected mice, and there was no evidence of glial activation or neuroinflammation. MA10 infection did not affect amyloid beta (Aβ) plaque volume or numbers in 5xFAD mice compared to uninfected mice. Spatial transcriptomics revealed altered expression of genes associated with homeostatic function in neurons, glia, and vascular endothelial cells.

**DISCUSSION:**

Collectively, these findings demonstrate that while MA10 infection did not affect AD neuropathology, there were numerous downstream effects on gene expression associated with resident central nervous system cell functions that may impact neurologic disease.

## Highlights

- SARS‐CoV‐2 infection has been associated with a spectrum of neurologic symptoms arguing for a potential role in affecting the severity of AD neuropathology and clinical disease.
- Infection of 5xFAD with a mouse‐adapted SARS‐CoV‐2 (MA10) did not affect Aβ plaque size/volume.
- MA10 infection altered homeostatic gene expression in neurons, glial cells, and endothelial cells in both 5xFAD and WT mice.

## 1 BACKGROUND

Alzheimer's disease (AD) is the most common cause of age‐related dementia, impairing memory and cognitive function. Pathology includes the accumulation of amyloid plaques and neurofibrillary tangles, accompanied by neuronal loss and neuroinflammation.^1^ AD is a multifactorial disorder influenced by several risk factors, including age,^2^ genetics,^3^ and environmental factors.^4^ Some studies suggest viral infections may accelerate disease onset or exacerbate existing pathology and clinical symptoms associated with neurodegenerative diseases, including AD,^5^ and other studies have implicated microbial pathogens in AD pathogenesis,^6^, ^7^ highlighting the potential contribution of infection to AD and related dementias.

Coronavirus disease 2019 (COVID‐19) is caused by severe acute respiratory syndrome coronavirus 2 (SARS‐CoV‐2) and rapidly developed into a global pandemic in 2020, affecting hundreds of millions of people worldwide. Infection presents with mild to moderate respiratory symptoms^8^; however, severe disease can result in acute respiratory distress syndrome (ARDS) and respiratory failure.^9^ Beyond respiratory problems, neurological symptoms and complications have been reported, including headache, dizziness, and olfactory and gustatory dysfunction.^10^ Severe COVID‐19 has also been associated with ischemic stroke, cerebrovascular disease, and seizures.^11^

Risk factors contributing to COVID‐19 severity and mortality include advanced age^12^ and pre‐existing dementia,^13^ and among patients with dementia, AD was the most prevalent cognitive impairment in those who died of COVID‐19.^14^ Additionally, COVID‐19 appears to impact brain structure and function, and some studies suggest that COVID‐19 may induce or accelerate neurodegenerative processes, including those related to dementia and AD.^15^ Furthermore, coronaviruses, such as SARS‐CoV‐1^16^ and, more recently, SARS‐CoV‐2,^17^ possess proteins with amyloidogenic properties that may promote amyloid aggregation. In addition, infection may alter resident central nervous system (CNS) cell function that can also contribute to alterations in neurologic dysfunction.

Neuroinflammation is a hallmark of many neurodegenerative diseases and is influenced by reactive microglia and astrocytes that lose their homeostatic functions, resulting in the release of proinflammatory cytokines that disrupt the blood–brain barrier (BBB), impair synaptic function, and promote neuronal death.^18^ In the context of COVID‐19, glial cells and neurons have been identified as targets of SARS‐CoV‐2 infection in patients and animal models,^19^, ^20^ although the virus is considered to have limited neuroinvasive capacity. *Post mortem* analyses of COVID‐19 patient brains have shown gliosis, axonal damage, and BBB disruption.^21^, ^22^ Similar findings have been made in animal models, where infection with SARS‐CoV‐2 or the mouse‐adapted SARS‐CoV‐2 MA10 strain leads to BBB disruption, gliosis, and the development of neuronal proteinopathies, even in the absence of direct CNS infection.^23^, ^24^ Cases where direct viral infiltration in the brain was observed also noted similar results along with increased expression of AD risk genes.^25^ Together, these findings suggest that COVID‐19‐associated neuroinflammation may contribute to hippocampal injury and neuronal and synaptic dysfunction.

In this study, we infected aged C57BL/6 wild‐type (WT) mice and 5xFAD mice with mouse‐adapted SARS‐CoV‐2 (SARS‐CoV‐2 MA10)^26^ to evaluate infection‐associated neurological changes and determine whether SARS‐CoV‐2 infection influences AD‐related neuropathology. The 5xFAD model is a widely used transgenic model of AD that overexpresses mutant human amyloid precursor protein and presenilin 1, resulting in early and progressive amyloid plaque deposition, neuroinflammation, synaptic dysfunction, and cognitive decline.^27^ Utilizing bulk RNA sequencing (RNA‐seq)and spatial transcriptomic analyses, we examined infection‐induced changes in neuronal, glial, and vascular pathways to better understand how SARS‐CoV‐2 infection may impact brain function in aging and AD‐relevant contexts and found that despite the absence of CNS infection alterations in neuronal, glial, and vascular pathways occurred, while amyloid beta (Aβ) plaque burden in 5xFAD mice remained unchanged.

**RESEARCH IN CONTEXT**

1. **Systematic review**: The authors reviewed the literature using traditional sources (e.g., PubMed), in addition to meeting abstracts and presentations. Although the impact of SARS‐CoV‐2 infection on the pathogenesis of AD has not been extensively studied, several publications using SARS‐CoV‐2 in mouse models of aging have highlighted its impact on neuroinflammation, neuronal health, and cognitive impairments. These publications have been appropriately considered and cited.
2. **Interpretation**: Our findings show that systemic infection with mouse‐adapted SARS‐CoV‐2 (MA10) does not impact Aβ plaque pathology in 5xFAD mouse model but does induce transcriptional changes across several cell types within the brain, such as microglia, neurons, and endothelial cells.
3. **Future directions**: This study highlights the transcriptional impact of peripheral mouse‐adapted SARS‐CoV‐2 infection in the brain. Future experiments will be required to determine whether these gene expression changes are sustained as the brain continues to age and translatable to brain samples from patients who survive SARS‐CoV‐2 infection. Furthermore, investigations into molecular mechanisms that modulate these SARS‐CoV‐2‐mediated gene expression changes will be beneficial to identify drug therapeutic targets that prevent the long‐term neurological consequences of COVID‐19.

## 2 METHODS

### 2.1 Viral infection of mice

Male and female 10 to 14 months old C57BL/6 WT and 5xFAD were used for these studies. Tg (APPSwFlLon, PSEN1*M146L*L286V) 6799Vas/ Mmjax RRID:MMRRC034848‐JAX was obtained from the Mutant Mouse Resource and Research Center (MMRRC) at The Jackson Laboratory, a National Institutes of Health‐funded strain repository, and was donated to the MMRRC by Robert Vassar, Ph.D., Northwestern University. Mouse‐adapted SARS‐Related Coronavirus 2 MA10 Variant (in isolate USA‐WA1/2020 backbone), Infectious Clone (ic2019‐nCoV MA10) in Calu‐3 Cells, NR‐55329, developed by Baric and colleagues,^28^ was used for the infection of the experimental mice. Age‐matched (10 to 14 months) 5xFAD and WT mice were intranasally inoculated with 5 × 10^3^ pfu of SARS‐CoV‐2 MA10 in 50 µL of 1× phosphate‐buffered saline (PBS) or sham inoculated. Infected and uninfected mice were monitored daily for signs of clinical illness.

### 2.2 Histology and immunohistochemical staining

Mice were euthanized at defined times post‐infection (p.i.), and brain and lungs were collected. Half brains were fixed in 4% PFA for 24 h at 4°C, then transferred into 30% sucrose in 1× PBS solution for cryoprotection for 48 h at 4°C, embedded in optimal cutting temperature (OCT) and 15‐µm sagittal sections were cut via cryostat (Thermo 95 664 OEC70 Micron HM525). Brain sections were desiccated overnight, and slides were rinsed with 1× Tris‐buffered saline (TBS) to remove residual OCT. Samples were blocked with 5% normal goat serum and 0.1% Triton‐X in TBS, followed by overnight incubation at 4°C with appropriate primary antibodies. On the second day, slides were treated with appropriate secondary antibodies following TBS rinsing. Amylo‐Glo staining (TR‐300‐AG; Biosensis) was performed according to the manufacturer's instructions. A complete list of antibodies and dilutions is provided in Table 1. Lungs were fixed and processed in the same manner as described above and stained with hematoxylin and eosin (H&E).

**TABLE 1:** Primary and secondary antibodies and associated information.

Primary antibody	Source	Catalog No.	Dilution	Secondary antibody	Source	Catalog No.	Dilution
IBA1	Wako	019‐19741	1:500	Goat anti‐rabbit IgG, AlexaFluor 488	Thermo Fisher Scientific	A11008	1:200
GFAP	Abcam	ab134436	1:1000	Goat anti‐chicken IgY Alexa Fluor 594	Abcam	ab150172	1:400
Mac2	Cedarlane	CL8942AP	1:500	Goat anti‐rat IgG Alexa Fluor 594	Abcam	ab150160	1:200
PSD95	Abcam	ab18258	1:500	Goat anti‐rabbit IgG, Alexa Fluor 488	Thermo Fisher Scientific	A11008	1:200
Synaptophysin	Sigma‐Aldrich	S5768	1:1000	Goat anti‐mouse IgG Alexa Fluor 555	Thermo Fisher Scientific	A21424	1:200
NeuN	Abcam	ab104225	1:100	Goat anti‐rabbit IgG, Alexa Fluor 488	Thermo Fisher Scientific	A11008	1:350
LAMP1	Abcam	ab25245	1:200	Goat anti‐rat IgG Alexa Fluor 594	Abcam	ab150160	1:200

Abbreviations: GFAP, glial fibrillary acidic protein; LAMP1, lysosome‐associated membrane protein 1; Mac2, Galectin 3; NeuN, neuronal nuclear antigen; PSD95, postsynaptic density protein 95.

### 2.3 Imaging and quantitative analysis

High‐resolution fluorescent images were obtained at 20× magnification using a Leica TCS SPE‐II confocal microscope and LAS‐X software. With confocal imaging, one field of view (FOV) per brain region was captured per mouse. Three brain regions per mouse were imaged: cortex (CRTX), dentate gyrus (DG), and subiculum (SUB). Cell counts and plaque burden were quantified using the spots module in Imaris. Volumetric measurements were automatically acquired using the surfaces module with confocal images of each brain region. Super‐Resolution Lattice Structure Illumination Microscopy (Lattice‐SIM) was performed using an Elyra 7 microscope system. The same three distinct anatomical regions (CRTX, DG, and SUB) per mouse were imaged at 40× magnification. Images were processed using ZEN SIM2 on the ZEN software before being analyzed on Imaris. For quantification of both pre‐ and postsynaptic puncta (synaptophysin and PSD95), the Imaris spots module was used to count the number of puncta for each z‐stack. Z‐stacks were 5 to 8 µm and the number of co‐localized spots was normalized to z‐stack volume. Overlapping puncta were defined as spots that were <0.2 µm apart.

### 2.4 RNA extraction and PCR

Mouse tissue was added to TRIzol and homogenized with the Bead Ruptor 12 (Omni International) and 1.4‐mm ceramic beads (Omni‐International, 19‐627). RNA was extracted via RNeasy minikit (Qiagen, 74106) using the “Purification of Total RNA, Including Small RNAs, from Animal Tissues” protocol, with Buffer RWT substituted by Buffer RW1. Library preparation, RNA‐seq, and read mapping analysis were performed by Novagene Co. Gene expression values were normalized into log_2_ FPKM (fragments per kilobase of transcript per million mapped reads). Heatmaps were created using Morpheus (Morpheus, https://software.broadinstitute.org/morpheus). Volcano plots were created using custom code in R. Differentially expressed genes (DEGs) were selected as significant with a log_2_ fold change (FC) > 0.5 and a false discovery rate (FDR) < 0.05. From the comparisons lists of genes of interest were chosen to plot a heatmap of their expression and a Gene Ontology (GO) term enrichment analysis using enrichR (https://amp.pharm.mssm.edu/Enrichr/).

### 2.5 cDNA synthesis and gene analysis

cDNA was made following the “First Strand cDNA Synthesis” protocol by New England Biolabs, using AMV Reverse Transcriptase (New England Biolabs, M0277L), Random Hexamers (Invitrogen, N8080127), RNAse inhibitor (New England BiolabsM0314L), and AMV Buffer (New England Biolabs B0277A). qPCRs were performed using the QIAquant 96 2 plex and iTaq Universal SYBER Green Supermix (BioRad, 1725120). Standard protocol for iTaq Universal SYBER GREEN Supermix was followed. Reactions were 10 µL, and the machine was set to run for one cycle (95°C for 3 min), followed by 40 cycles (95°C for 15 s, then 58°C for 30 s). Sequences for mouse GAPDH, forward primer: AACTTTGGCATTGTGGAAGG, reverse primer: GGATGCAGGGATGATGTTCT; SARS‐CoV‐2 Nucleocapsid primers were purchased from Integrated DNA Technologies, forward (IDT, 10006821) and reverse (IDT, 10006822).

### 2.6 Enzyme‐linked immunosorbent assay (ELISA)

SARS‐CoV‐2 spike protein‐specific antibodies in the plasma of infected mice were measured by ELISA. Further, 96‐well plates were coated with 100 ng per well of Recombinant SARS‐CoV (2019‐nCoV) Spike S1+S2 ECD‐His Recombinant Protein No. 40589‐V08B1 from Sinobiological and incubated overnight at 4°C. Plates were then washed three times with 1× PBS and blocked with 3% bovine serum albumin in 1× PBS for 1 h at room temperature (RT). Following blocking, serial dilutions of mouse plasma (100 µL/well) were added and incubated for 2 h at RT. Bound antibodies were detected using horseradish peroxidase‐conjugated goat anti‐Mouse IgG (H+L) (Catalog No.: 31430 from Invitrogen) and developed with Tetramethylbenzidine (TMB) substrate (Thermo Fisher Scientific, Reference No.: 34028). The reaction then stopped with Stop reagent for TMB substrate (Catalog No.: S514‐100ML from Millipore). Plates were read with the ELISA reader at 450 nm.

### 2.7 Cytokine profiling using Luminex platform

One aliquot of frozen plasma samples from mice was thawed to perform cytokine profiling using the Luminex Intelliflex platform. We first performed a titration assay, and three samples from each cohort were used and diluted at 1:25, 1:50, and 1:100. Then these three dilutions and a neat control were screened using the MILLIPLEX MAP mouse kit “Milliplex Mouse Cytokine/Chemokine MAGNETIC BEAD Premixed 32 Plex Kit,” which was the same kit intended for general assay. After measuring analytes and comparing concentrations, neat samples were chosen as the best representatives of concentration values in the samples measured. Hence, plasma samples were used in a neat state (no dilution) in the general assay, and 10 µL of each sample was assessed in a 384‐well plate for cytokine profiling. The Milliplex Mouse Cytokine/Chemokine MAGNETIC BEAD Premixed 32 Plex Kit (G‐CSF, EOTAXIN, GMCSF, IFNY, IL1a, Il1b, IL2, IL4, IL3, IL5, IL6, IL7, IL9, IL10, IL12P40, IL12P70, LIF, IL13, LIX, IL15, IL17, IP10, KC, MCP1, MIP1A, MIP1B, MCWF, MIP2, MIG, RANTES, VEGF, TNFA) was used to measure plasma cytokine and chemokines. To adjust the manufacturer's protocol to our 384‐well plate format, all other reagents, including antibodies, magnetic beads, and detection reagents, were used at 10 µL. Outcomes from wells with <50 beads for each analyte were excluded from analysis. Plates were read using an xMAP INTELLIFLEX System (Luminex). Quality control was performed on cytokine quantification data using the Belysa Immunoassay Curve Fitting Software (Millipore). This consisted of evaluating standard curves for all 32 analytes and quality control samples (provided in each kit as QC1 and QC2) measured in the experiment. The interpolated concentrations of the plasma samples were exported in an Excel file format for statistical analysis. Graph Pad Prism version 10.5 was used for analysis and image generation. For statistical analysis a two‐way ANOVA was fit to model the log expression of each cytokine. Significant differences were evaluated by group using the overall F test followed by pairwise comparisons under Tukey's Honestly Significant Difference procedure.

### 2.8 Spatial transcriptomic analysis

Formalin‐fixed brain hemispheres were embedded in OCT compound (Sakura Fintek, 4583) and stored frozen at −80°C. Sagittal sections were cut at 10 µm the day before starting the protocol on a Leica cryostat (LeicaBiosystems, CM1950). Six brain sections were mounted onto VWR Superfrost Plus slides (Avantor, 48311‐703), allowed to dry at RT for 15 min to promote tissue adherence, and stored overnight at −80°C. Tissue was processed in accordance with the Nanostring CosMx fresh‐frozen slide preparation manual for RNA (NanoString University). On day 1 of the slide preparation protocol, slides were removed from −80°C and baked at 60°C for 1 h. Slides were then processed for CosMx: three 5‐min washes in 1× PBS, one 2‐min wash in 4% sodium dodecyl sulfate (Thermo Fisher Scientific, AM9822): three 5‐min washes in 1× PBS, one 5‐min wash in 50% ethanol, one 5‐min wash in 70% ethanol, and two 5‐min washes in 100% ethanol before allowing slides to air dry at RT for 10 min. A pressure cooker was used to maintain slides at 100°C for 15 min in preheated 1× CosMx Target Retrieval Solution (Nanostring) for antigen retrieval. Slides were then cooled in DEPC‐treated water (Thermo Fisher Scientific, AM9922) for 15 s, transferred to 100% ethanol for 3 min, and then isolated at RT for 30 min to air dry. For effective tissue permeabilization, slides were transferred to a digestion buffer (3 µg/mL Proteinase K in 1× PBS; Nanostring) to incubate, then washed twice for 5 min in 1× PBS. Fiducials for imaging were diluted to 0.00015% in 2× SSC‐T (Saline Sodium Citrate with Tween20) and left for 5 min on slides to incubate. Slides were shielded from light following fiducial application. A 10% neutral buffered formalin (NBF) solution (Catalog No.: 15740) was applied for 1 min to post‐fix tissues. Slides were then washed for 5 min with NBF Stop Buffer (0.1 M Tris‐Glycine Buffer, Catalog No.: 15740) twice and washed for 5 min with 1× PBS. An NHS‐Acetate (100 mM; Catalog No.: 26777) solution was then administered to the slides and incubated for 15 min at RT. Slides were then washed twice for 5 min with 2× SSC and incubated in modified 1000‐plex Mouse Neuroscience panel (Nanostring) with the addition of an rRNA segmentation marker for in situ hybridization in a 37°C hybridization oven overnight for 16 to 18 h. On day 2 of slide preparation, slides were placed in preheated stringent wash solution (50% deionized formamide [Catalog No.: AM9342]) in 2× saline‐sodium citrate [SSC; Catalog No.: AM9763]) at 37°C twice for 25 min each. Slides were then washed in 2× SSC twice for 2 min each. Slides were then incubated with DAPI nuclear stain for 15 min, washed for 5 min with 1× PBS, incubated with histone and glial fibrillary acidic protein (GFAP) cell segmentation markers for 1 h, and washed three times for 5 min each in 1× PBS. Adhesive flow cells (Nanostring) were placed in each slide to create a fluidic chamber for spatial imaging. Slides were loaded into the CosMx instrument and automatically processed. On each slide, approximately 600 total FOVs (∼100 FOVs per brain section) were chosen that captured hippocampal, corpus callosum, upper thalamic, upper caudate, and cortical regions for each of the six sections. Imaging progressed for approximately 7 days and data were uploaded automatically to the Nanostring AtoMx online platform. Preprocessed data were exported from AtoMx as a Seurat object for analysis. Spatial transcriptomics datasets were processed with R 4.3.1 software, as previously described. Principal component analysis (PCA) and Uniform Manifold Approximation and Projection (UMAP) analysis were performed to reduce dataset dimensionality and visualize clustering. Data‐driven clustering at 1.0 resolution yielded 46 clusters. Clusters were manually annotated based on transcript expression of known marker genes and location in XY space. The detection of oligodendrocyte‐associated transcripts, for example, *Mbp* in glial clusters, can result in “doublets” and reflect the technical limits of spatial segmentation in highly myelinated regions, a common observation in high‐resolution spatial platforms. Cell proportion plots were generated by first visualizing the number of cells in each cell type, then scaling relative to (1) normalized percentages per group, calculated by dividing the number of cells in each cell type‐group pair by the total number of cells, and (2) dividing by the sum of the proportions across the cell type to account for differences in sample sizes. MAST was used on scaled expression data to perform differential gene expression analysis per cell type between groups to compute the average difference, defined as the difference in log‐scaled average expression between the two groups for each non‐specific cell type. The absolute log_2_ FC values of all genes with statistically significant gene (i.e., *p_adj_* < 0.05) differential expression patterns between two groups were summed to calculate the DEG scores between group pairs for each subcluster. ggplot2 3.4.4174 was used to generate data visualizations.

### 2.9 Statistical analysis

GraphPad Prism was used to perform statistical analysis on immunohistochemical data. Data were analyzed using two‐way ANOVA, and Tukey's post hoc tests were used to evaluate biologically relevant interactions. Each dataset was examined for sex differences, and if none were found, both male and female data were combined; a *p* value of < 0.05 was considered statistically significant. Data for each experiment are presented as mean ± SEM.

## 3 RESULTS

### 3.1 MA10 infection of WT and 5xFAD mice

**FIGURE 1:**
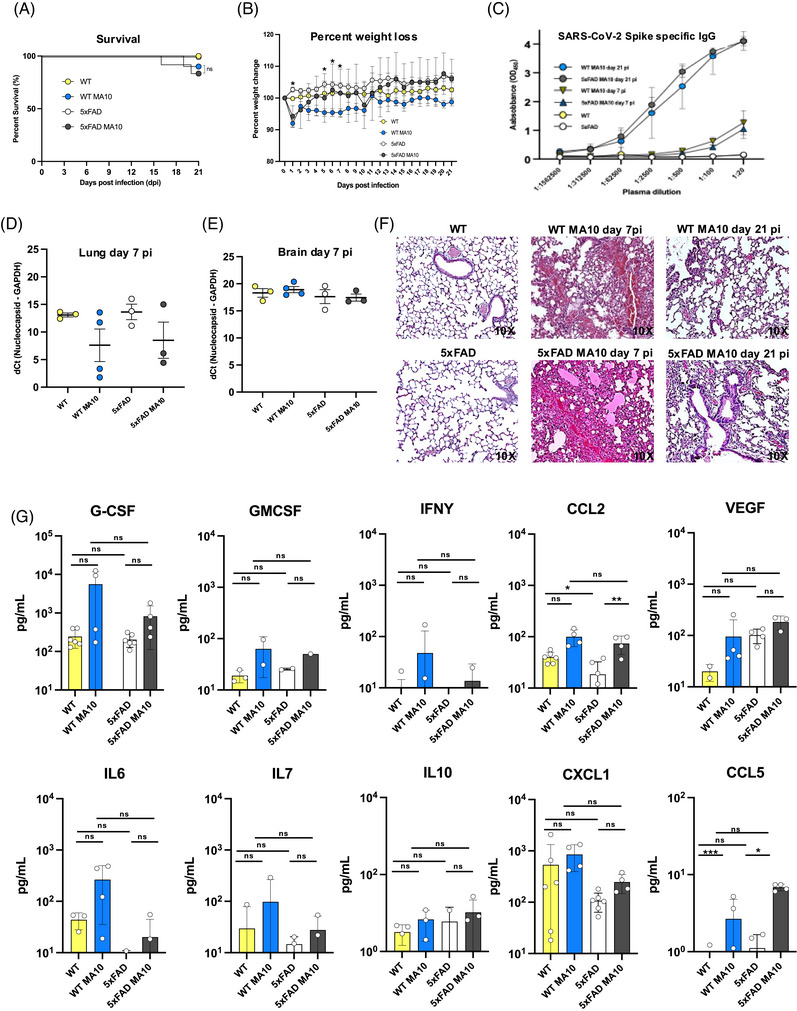
MA10 infection elicits mild disease in infected mice, and genotype does not affect mortality. (A) Percent survival of uninfected wildtype (WT) and 5xFAD compared to MA10‐infected WT and C57BL/6 mice. (B) Average body weight changes each day p.i. normalized to the body weight on the day of infection, with indicating significance (*p* < 0.05) between MA10‐infected WT and uninfected WT. (C) Neutralizing IgG anti‐spike antibodies were present in the plasma of MA10‐infected mice at days 7 and 21 p.i. as assessed by ELISA. RT–qPCR was performed on (D) lung and (E) brain homogenates at day 7 p.i. to quantify MA10 nucleocapsid (N) gene expression. The data are presented as dCt values, calculated by subtracting the Ct of the housekeeping gene GAPDH from the Ct of the viral nucleocapsid (N) gene. Lower dCt values indicate higher viral RNA abundance. Each dot represents an individual mouse; bars represent the mean ± SEM. (F) Representative H&E‐stained lung images at 10× magnification from uninfected and MA10‐infected WT and 5xFAD mice at both 7 and 21 days p.i. are shown. Pathological features associated with infection included airway edema, vascular congestion and intra‐alveolar hemorrhage, peri‐bronchiolar lymphocytic cuffing, and interstitial vascular congestion and lymphocytic infiltrates. (G) Cytokine concentrations in plasma were measured using a multiplex bead‐based immunoassay on the Luminex platform. Data for individual cytokines are shown, with each dot representing an individual mouse and bars indicating the mean ± SEM. *p < 0.05 between MA10‐infected WT and uninfected WT.

C57BL/6 (WT) and 5xFAD mice (10 to 14 months of age) were intranasally (i.n.) infected with 5 × 10^3^ pfu of MA10. Animals were sacrificed at days 7 and 21 (p.i.) and lungs and brains harvested to assess tissue pathology, presence of viral RNA, and effects on AD‐associated neuropathology. By 4 months of age, 5xFAD mice develop Aβ plaques in the brain, which increase in number and size over time, plateauing between 10 and 12 months of age.^27^, ^29^ By day 21 p.i., there was no significant difference in mortality, with ∼20% of infected 5xFAD mice and ∼15% of infected WT mice dying in response to infection (Figure 1A). Daily weighing of mice showed MA10‐infected WT mice exhibited significant weight loss compared to their uninfected controls only at 1 and 5 to 7 p.i., while MA10‐infected 5xFAD mice did not demonstrate significant weight loss in comparison to their uninfected controls (Figure 1B). Spike‐specific IgG antibodies were detected at days 7 and 21 p.i. in both WT and 5xFAD mice, confirming infection with MA10 compared to sham‐infected mice (Figure 1C). Viral RNA was examined via qPCR in lungs and brains of infected mice at 7 days p.i. Low levels of viral RNA were present at day 7 p.i. within the lungs (Figure 1D). No viral RNA was detected in the brains at 7 days p.i. (Figure 1E), consistent with previous reports indicating MA10 does not readily infect the CNS.^23^ There were no significant differences between uninfected and infected cohorts, and any Ct values yielded from infected cohorts in qPCR were assumed to be background, as they were like those of uninfected/control mice. H&E staining of lungs of infected WT and 5xFAD mice at day 7 p.i. demonstrated both alveolar and interstitial lesions, with alveolar hemorrhage and edema, interstitial congestion, and lymphocytic infiltrates within the lungs and by day 21 p.i. There was still evidence of inflammation in both MA10‐infected WT and 5xFAD mice, although this was reduced in comparison to day 7 p.i. (Figure 1F). Examination of proinflammatory cytokines and chemokines within the blood of MA10‐infected WT and 5xFAD mice at day 7 p.i. showed a trend toward increased levels of proinflammatory cytokines in infected mice compared to their controls; however, the differences did not reach statistical significance for all markers. MA10 infection did result in a significant increase in CCL2 and CCL5 in both WT and 5xFAD mice compared to uninfected controls (Figure 1G).

### 3.2 Microglia and astrocytes in MA10‐infected mice do not exhibit changes in numbers or volume

**FIGURE 2:**
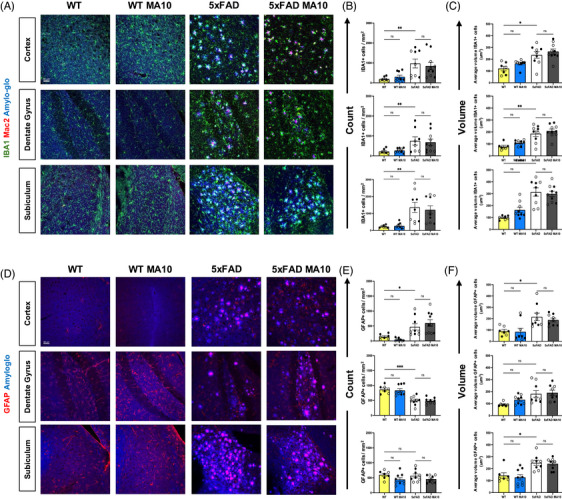
Effect of MA10 infection on glial activation in WT and 5xFAD mouse brains. Brains from uninfected and MA10‐infected WT and 5xFAD mice at day 21 p.i. were immunostained for IBA1 and MAC2 to assess changes in microglia and infiltrating monocytes/macrophages, respectively, and GFAP to detect astrocytes. Dense‐core Aβ plaques were stained via Amylo‐Glo. (A) Representative images (20× magnification) from brains isolated from uninfected and infected WT and 5xFAD mice showing cortex (CRTX), dentate gyrus (DG), and subiculum (SUB) stained for microglia (IBA1), macrophages (MAC2), and Amylo‐Glo. (B) Average IBA1+ cells per square millimeter in CRTX (upper graph), DG (middle graph) and SUB (bottom graph) from experimental mice. (C) IBA1 average volume per um^3^ in CRTX (upper graph), DG (middle graph) and SUB (bottom graph) from experimental mice. (D) Representative images from brains isolated from uninfected and infected WT and 5xFAD showing GFAP expression and Aβ plaques. Average GFAP+ cells per mm^2^ (E) and average volume per µm^3^ (F) in CRTX (upper graphs), DG (middle graphs), and SUB (bottom graphs). Immunohistological data were analyzed using two‐way ANOVA. Tukey's post‐hoc test was employed to examine biologically relevant interactions. Female and male mice are indicated by open or closed circles, respectively. Data are represented as MEAN ± SEM. *p < 0.05, **p < 0.01, ***p < 0.001, ****p < 0.0001. Scale bar in (A) = 50 µm.

Brains from MA10‐infected and control mice were immunofluorescently labeled for microglia (IBA1) and astrocytes (GFAP) to determine whether there was an increase in glial activation in response to MA10 infection at day 21 p.i. We elected to examine the CRTX, DG, and SUB, as previous studies of 5xFAD mice indicated changes in AD‐associated pathology in these regions.^29^ As a metric of cell activation, we measured either GFAP+ or IBA1 cell numbers and volume in control and MA10‐infected WT and 5xFAD mice. Representative images of IBA1+ microglia and Mac2+ monocytes, along with Amylo‐Glo counterstain for Aβ plaques (5xFAD mice), were acquired within the CRTX, DG, and SUB from uninfected and MA10‐infected WT and 5xFAD mice are shown (Figure 2A). Quantification of microglia numbers (Figure 2B), and volume (Figure 2C) does not indicate significant changes in MA10‐infected WT or 5xFAD mice compared to their uninfected controls. There was an increase in microglia number and volume in both uninfected and infected 5xFAD mice compared to uninfected and infected WT mice in all brain regions examined (Figure 2C).

MAC2 is a unique marker for peripheral monocyte‐derived macrophages that allows discrimination between these cells and microglia.^30^ Immunostaining and quantification do not indicate changes in MAC2‐positive cell number or volume within the CRTX, DG, or SUB as a result of MA10 infection in either WT or 5xFAD mice (Figure S1). There were increased numbers (*p* < 0.0001) of MAC2‐positive cells in the brains of uninfected 5xFAD mice compared to uninfected WT mice, reflective of the neuroinflammation occurring within the brains of the 5xFAD mice as a result of ongoing AD neuropathology and Aβ plaque accumulation (Figure S1). Although MAC2 can be expressed by some activated microglial subsets, our spatial transcriptomic data did not identify a unique MAC2‐high myeloid population distinct from the resident clusters. Moreover, other studies have demonstrated distinct macrophage populations that were separate from resident microglia, and our findings support these observations.^31^ Immunofluorescent staining of GFAP+ astrocytes in uninfected and MA10‐infected WT and 5xFAD mice, along with Amylo‐Glo staining of Aβ plaques in control and MA10‐infected 5xFAD mice within the CRTX revealed no differences in GFAP expression (Figure 2D). Quantification of both astrocyte numbers and volume revealed no differences between control and infected WT and 5xFAD mice, indicating that infection did not result in increased astrocyte activation at 21 days p.i. (Figure 2E,F). There were increased numbers of GFAP+ cells within the CRTX of uninfected 5xFAD mice compared to uninfected WT mice, while volume of GFAP+ cells was increased in both the CRTX and SUB in uninfected 5xFAD mice compared to uninfected WT mice (Figure 2F). GFAP numbers were reduced in the DG of uninfected 5xFAD mice compared to uninfected WT mice (Figure 2F). Overall, these findings indicate that MA10 infection of either WT or 5xFAD does not result in sustained activation of either microglia or astrocytes by 21 days p.i.

### 3.3 Dystrophic neurite and Aβ plaque burden in MA10‐infected mice

**FIGURE 3:**
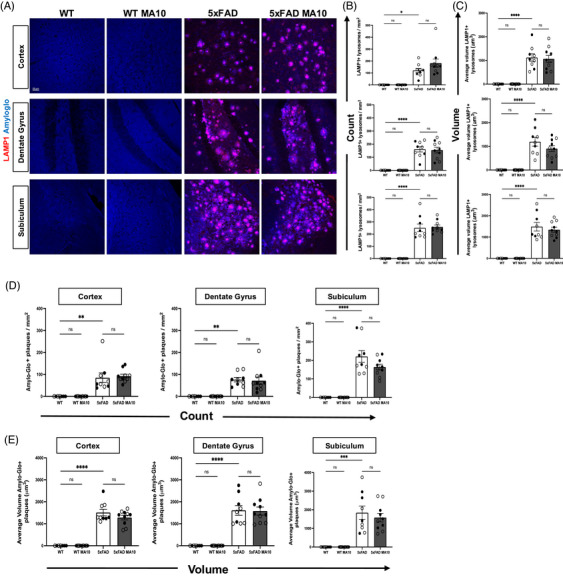
MA10 infection does not affect dystrophic neurites or Aβ plaques in 5xFAD mice. Brains from uninfected and MA10‐infected mice at 21 days after infection were immunostained with LAMP1 to visualize dystrophic neurites and with Amylo‐Glo to detect dense‐core Aβ plaques. (A) Representative images of LAMP1 and Amylo‐Glo staining in the CRTX, DG, and SUB of uninfected and infected WT and 5xFAD mice. (B) Quantification of LAMP+ lysosome density (B) and volume (C) showed no differences in MA10‐infected WT or 5xFAD mice compared to uninfected controls. Aβ plaque burden was determined through quantifying Amylo‐Glo^+^ plaque number as shown in (D) and summarizes the average volume of these plaques (E). Immunohistological quantifications were analyzed using two‐way ANOVA followed by Tukey's post hoc test. Female and male mice are represented by open and closed circles, respectively. Data are presented as mean ± SEM; *p ≤ 0.05, ****p ≤ 0.0001. Scale bar in (A) = 50 µm.

Dense‐core Aβ plaques, a key feature of AD pathology, are surrounded by dystrophic neurites, which are abnormal swollen neuritic processes that can be detected by immunostaining for lysosome‐associated membrane protein 1 (LAMP1).^29^ Representative images of LAMP1+ lysosomes with Amylo‐Glo‐positive Aβ plaques were acquired for the CRTX, DG, and SUB (Figure 3A). Dystrophic neurites and Aβ plaque pathology are only present in 5xFAD, and infection does not affect or induce these pathologic features in WT mice (Figure 3A). Immunostaining and quantification do not indicate changes in count or volume of dystrophic neurites in response to MA10 infection of either WT or 5xFAD mice compared to uninfected controls in any of the brain regions examined in either WT or 5xFAD mice (Figure 3B,C). There were no differences in either Aβ plaque count or volume in MA10‐infected 5xFAD mice compared to uninfected 5xFAD mice (Figure 3D,E). Synaptic puncta were next quantified via co‐localization of the presynaptic marker, synaptophysin, and postsynaptic marker PSD95 to assess effects of MA10 infection on neuronal dysfunction. Representative images of synaptophysin puncta, and PSD95 positive puncta were acquired (Figure S2A). Immunostaining and quantification did not indicate changes in synapse numbers in the brains of either WT or 5xFAD mice at 21 days p.i. (Figure S2B).

### 3.4 Bulk RNA‐seq of brains from MA10‐infected mice

**FIGURE 4:**
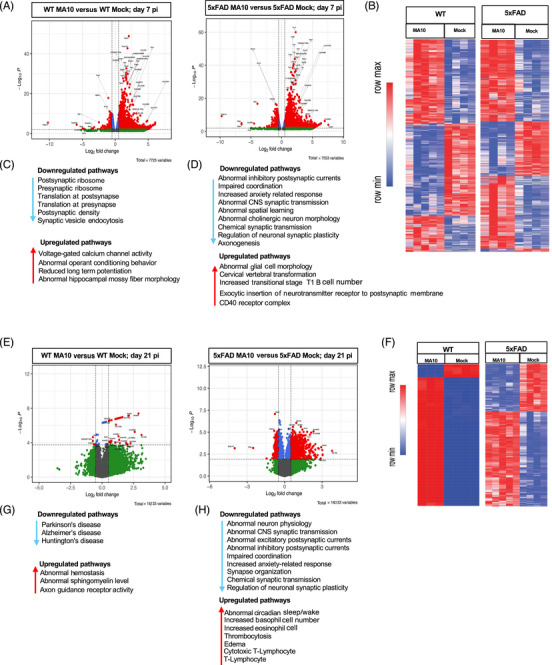
SARS2 MA10 infection in brains of WT and 5xFAD mice is associated with differential expression of genes involved in regulation of synaptic activity on days 7 and 21 p.i. RNA sequencing was performed on brains from MA10 infected and control WT and 5xFAD male mice at 7 days p.i. (A) Volcano plot of differentially expressed genes (DEGs) displaying fold change of genes (log_2_ scale) and *p* values. (B) Heatmap of selected (FC = 0.5, FDR < 0.05) DEGs from MA10‐infected WT and 5xFAD and uninfected (mock) controls at day 7 p.i. Enriched pathways of interest examined via Gene Ontology (GO) expressed in (C) MA10‐infected WT and (D) 5xFAD mice showing down‐ and upregulated pathways at day 7 p.i. Volcano plot of DEGs displaying fold change of genes (log_2_ scale) and *p* values in (E) MA10‐infected WT and 5xFAD mice at day 21 p.i. (F) Heatmap of selected (FC = 0.5, FDR < 0.05) DEGs from MA10‐infected WT and 5xFAD and uninfected (mock) controls at day 21 p.i. Enriched pathways of interest examined via GO expressed in (G) MA10‐infected WT and (H) 5xFAD mice showing down‐ and upregulated pathways at day 21 p.i.

Bulk RNA‐seq of DEGs was performed on the brains of experimental mice at day 7 and 21 p.i. Volcano plots of brain gene expression in MA10‐infected and control WT and 5xFAD at days 7 (Figure 4A) and 21 p.i. (Figure 4E) were plotted. DEGs were selected as significant with a log_2_ FC > 0.5 and FDR < 0.05. On both days 7 and 21 p.i., infected WT and 5xFAD groups showed differential expression of genes compared to control groups. At day 7 p.i., both groups exhibited a strong transcriptional response, with numerous genes significantly upregulated (Figure 4A). By day 21 p.i., MA10‐infected WT mice showed fewer numbers of DEGs compared to MA10‐infected 5xFAD mice, suggesting a more persistent molecular response in MA10‐infected 5xFAD mice (Figure 4E). Heatmaps were created to compare significant DEGs from control and MA10‐infected WT and 5xFAD mice. DEGs were selected as significant if log_2_ FC was greater than 0.5 and FDR/adjusted *p* value was less than 0.05 (FC > 0.5, FDR < 0.05). Genes that met such criteria were then used to plot heatmaps of their expression from the brains of experimental mice at days 7 (Figure 4B) and 21 p.i. (Figure 4F). GO analysis of MA10‐infected WT mice at day 7 p.i. revealed increased expression of pathways associated with voltage‐gated calcium channel activity, abnormal operant conditioning, reduced long‐term potentiation (LTP), and abnormal hippocampal mossy fiber morphology (Figure 4C). In the MA10‐infected 5xFAD mice at day 7 p.i. analysis indicated changes in pathways associated with abnormal postsynaptic currents, synaptic transmission, neuron morphology, and spatial learning, as well as impaired coordination and increased anxiety‐related responses. Additionally, there are changes in pathways associated with synaptic transmission and plasticity as well as axonogenesis (Figure 4D). Brains of both the WT and 5xFAD groups did indicate lasting changes in gene expression (Figure 4E); however, heatmap analysis indicated that the MA10‐infected 5xFAD group had dramatically more lasting DEGs in comparison to the MA10‐infected WT group (Figure 4F). Moreover, when examining pathways of interest, MA10‐infected WT mice showed only one identified downregulated gene (*UQCRFS1*) that is associated with AD^32^ (Figure 4G). Analysis of the 5xFAD group at 21 days p.i. indicated changes of many more pathways, most of which were associated with neuronal and synaptic dysfunction, impaired coordination, and anxiety‐related responses (Figure 4H). Furthermore, the brains of MA10‐infected WT mice indicated changes in gene expression in pathways associated with abnormal sphingomyelin levels and axon guidance receptor activity (Figure 4G), while the brains of MA10‐infected 5xFAD mice indicated changes in pathways associated with edema and abnormal circadian cycles (Figure 4H).

### 3.5 Altered gene expression in resident CNS cells following MA10 infection of WT and 5xFAD mice

**FIGURE 5:**
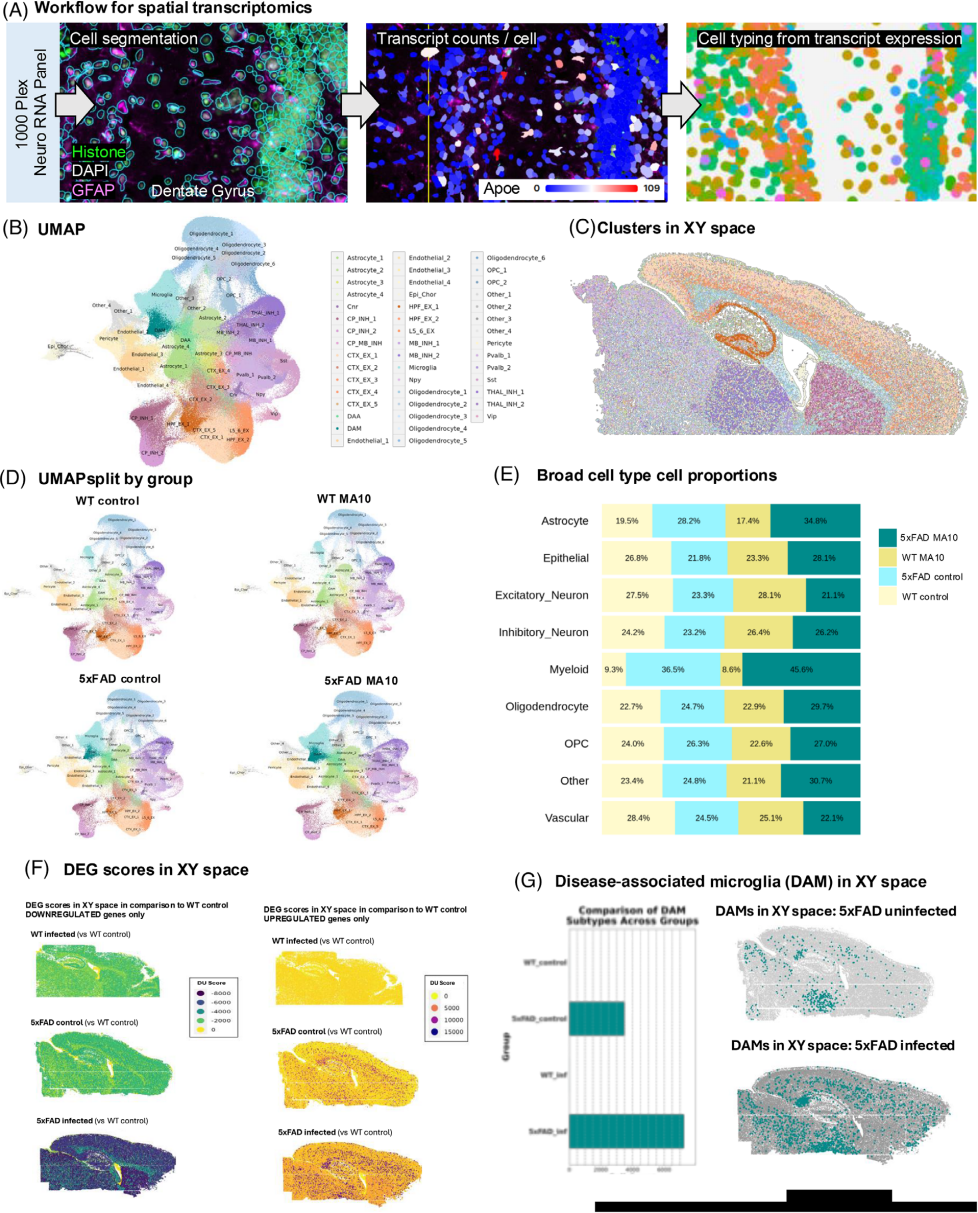
Spatial transcriptomic analysis of MA10‐infected WT and 5xFAD mouse brain. (A) Experimental workflow for targeted 1000‐plex single‐cell spatial transcriptomics. Fields of view (FOVs) were selected in hippocampal, corpus callosum, upper thalamic, upper caudate, and cortical regions for each of the six sections then imaged with DNA, rRNA, histone, and GFAP markers for cell segmentation. Transcript counts for each gene were acquired per cell. (B) Uniform Manifold Approximation and Projection (UMAP) of 787,580 cells across six brains were captured with a mean transcript count of ∼600 per cell. Unbiased cell clustering identified 46 transcriptionally distinct cell clusters, which were annotated with a combination of automated and manual approaches with reference to Allen Brain Atlas single‐cell RNA sequencing cell types, gene expression, and anatomical location in space. (C) Forty‐six clusters plotted in XY space. (D) Separation of clusters by group (WT control, MA10‐infected WT, 5xFAD control, MA10‐infected 5xFAD). (E) Proportional distributions across experimental groups. Bar plots display relative abundance of each cluster in each group. (F) Downregulated DEG scores in XY space in comparison to WT control (left panel), upregulated DEGs (right panel). (G) DAM cell counts across groups (left panel), distribution of DAMs plotted in XY space in uninfected and MA10‐infected 5xFAD.

WT and 5xFAD MA10‐infected and uninfected control brains were sectioned and processed for spatial transcriptomic analysis and analyzed for 1000 genes using the Nanostring CosMx Spatial Molecular Imager platform (*n* = 3 per group, 1216 total FOVs, ∼100 FOVs per brain section) (Figure 5A; examples of cell segmentation in Figure S3). A total of 787,580 cells were captured with a mean transcript count of ∼600 per cell. Unbiased cell clustering identified 46 transcriptionally distinct cell clusters (Figure 5B and Figure S4A,B), which were manually annotated based on gene expression and location. Projection of clusters in XY space revealed distinct localization to specific anatomical regions (Figure 5C and Figure S5). Separation of cell clusters by group (WT control, WT MA10 infected, 5xFAD control, 5xFAD MA10 infected) revealed distribution of distinct cell populations within the brains of each experimental group (Figure 5D). Clusters from the same broad cellular subtype were then combined into broad cell types (i.e., Astrocyte 1 and Astrocyte 2, for example, were assigned to the astrocyte cell type) for subsequent analyses. The proportion of cells in each major cluster from experimental groups was then plotted per genotype (Figure 5E). To broadly assess overall deviation from WT in a given group, DEG scores were calculated for each cellular subtype. To calculate DEG scores for each group, the log_2_FC values were summed for DEGs that were significantly up‐ or downregulated in comparison to WT (i.e., *p_adj_* ≤ 0.05), yielding DEG scores for each subtype. Subtypes were then plotted in XY space colored by DEG score for up‐ and downregulated genes to give a visual impression of the spatial distribution of DEGs and overall degree of deviation from WT control in the three experimental groups (Figure 5F). As expected, the 5xFAD MA10‐infected brains had the highest up‐ and downregulated DEG scores, indicative of broad deviation from WT gene expression patterns. As disease‐associated microglia (DAMs) are considered important in Aβ pathology in 5xFAD‐infected mice,^33^ we next plotted the DAM cluster in XY space, revealing that DAMs were distributed in both uninfected and MA10‐infected 5xFAD mice in plaque‐laden areas, that is, the SUBof the hippocampus, throughout the CRTX (Figure 5G).

### 3.6 MA10 infection alters gene expression in glial cells and neurons

**FIGURE 6:**
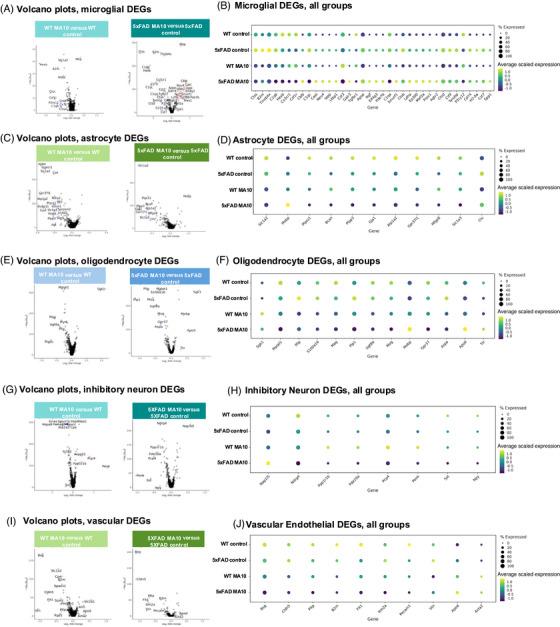
SARS2 MA10 infection leads to changes in gene expression within resident CNS cells, as revealed by spatial transcriptomic analysis of WT and 5xFAD mouse brains. Volcano plots of DEGs between each of the following broad cell types: (A) microglia, (C) astrocyte, (E) oligodendrocyte, (G) inhibitory neuron, and (I) vascular cells of both MA10‐infected WT versus WT control (left panel) and MA10‐infected 5xFAD MA10 versus 5xFAD control (right panel). (B), (D), (F), (H), and (J) represent corresponding dot plots showing expression of individual genes across experimental groups (WT control, 5xFAD control, WT MA10, and 5xFAD MA10). Dot size represents percentage of cells expressing gene, while color indicates average scaled expression level.

Differential gene expression analysis revealed that MA10 infection led to the appearance of DEGs within broad cell types in both WT and 5xFAD mice, including microglia, astrocytes, oligodendrocytes, inhibitory neurons, and vascular endothelial cells (Figure 6A–J). Many DEGs were identified between infected and uninfected control brains with infection in both 5xFAD and WT mice. In both mice, microglia demonstrated loss of numerous homeostatic signature genes^34^ (i.e., *Hexb*, *Csf1r*, *P2ry12*, *Cx3cr1*) (Figure 6A,B). Astrocytes indicated a downregulation of *Slc1a2*, *Gja1*, *Plpp3*, *Atp1a2*, and *Ptprz1*, genes identified to be involved in astrocytic functions that support neuronal communication and homeostasis^35^, ^36^, ^37^ (Figure 6C,D). Furthermore, oligodendrocytes showed decreased expression of genes related to myelination, lipid metabolism, and neuroprotection (*Malat1*, *Mag*, *Ugt8a*, and *Plp*)^38^, ^39^, ^40^ as well as an increased expression in genes related to cellular stress responses (*Sgk1*)^41^ (Figure 6E,F). Other cell types, such as inhibitory neurons, exhibited a downregulation of genes associated with neurotropic support (*Ndrg4*, *Sst*, and *Npy*)^42^, ^43^, ^44^ (Figure 6G,H), while vascular endothelial cells indicated consistent changes across genotypes in both the downregulation of genes associated with BBB integrity (*Bsg*, *Cldn5*, and *Pecam1*)^45^, ^46^ as well as the upregulation of genes associated with other vascular adaptations (*Apod* and *Acta2*)^47^, ^48^ (Figure 6I,J).

WT brains demonstrated a greater number of significant DEGs and/or a greater average difference than 5xFAD brains between infected and uninfected controls in the majority of broad cell types. Microglia represented a notable exception in that they were far more affected in 5xFAD brains than WT brains following infection; this is most likely because 5xFAD microglia are already highly dysregulated as a result of Aβ accumulation over time. While both WT and 5xFAD genotypes showed loss of homeostatic microglial markers after infection in comparison to their respective uninfected controls, the average difference (log_2_FC) and significance (*p_adj_*) in 5xFAD microglia were higher than in WT. In addition to conservation of the majority of downregulated genes from WTs, 5xFAD microglia also had a number of upregulated genes that were not present in WTs, including *Cblb*, *Cast*, *Neu4*, *Iapp*, *Apoe*, *Ltbp1*, *Eif4g3*, *Ngf*, and *Smurf1*, among others (Figure 6B), indicating a further loss of microglial homeostatic functions, such as survival, communication, migration, and phagocytic capacity to an even more reactive state, suggesting that 5xFAD microglia are more affected by MA10 infection than WT microglia at the transcriptomic level. A complete comparison of DEGs in MA10‐infected WT and 5xFAD mice compared to uninfected controls is provided in Figures S6 and S7, respectively.

### 3.7 Comparing effects of Aβ pathology and MA10 infection on cell‐type‐specific differential gene expression

**FIGURE 7:**
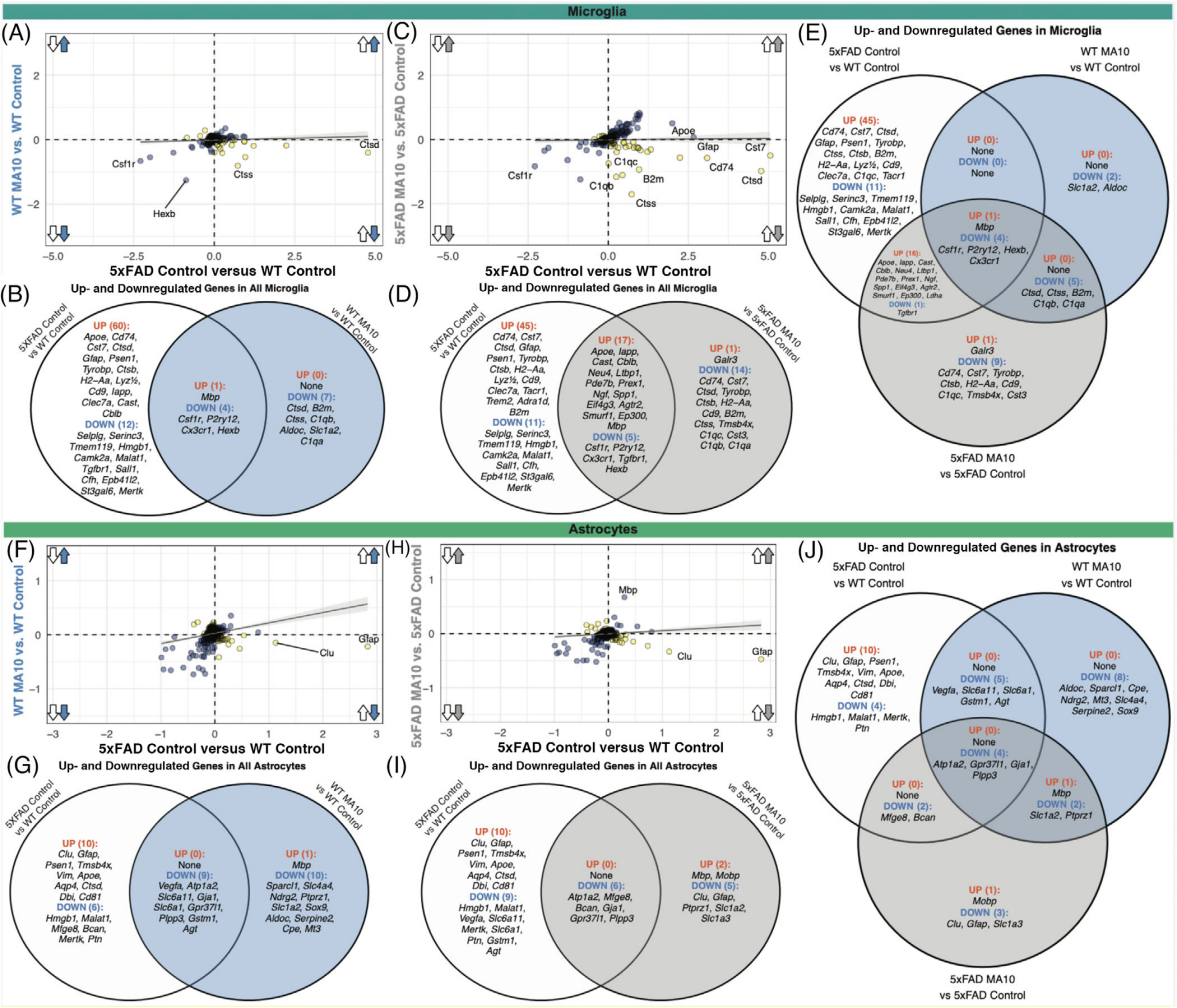
Microglia and astrocytes have differential responses to the presence of amyloid pathology, peripheral MA10 infection, or both concurrently. (A) Scatterplot of average difference of all microglia for all significant genes (*p_adj_* < 0.05) between 5xFAD control versus WT control (*x*‐axis, only amyloid pathology) and WT MA10 versus control WT (*y*‐axis, only MA10 infection) comparisons. Arrows indicate direction of dysregulation for each comparison. Directly correlated genes (blue) occur in the same direction for both comparisons (i.e., both upregulated or both downregulated), while inversely correlated genes (orange) occur in opposite directions for each comparison. Linear regression line demonstrates the relationship between the two comparisons. (B) Venn diagram depicting significant up‐ and downregulated genes unique to 5xFAD control versus WT control (left, white) and WT MA10 versus WT control (right, blue), while demonstrating up‐ and downregulated genes commonly shared across the two comparisons (middle). (C) Scatterplot of average difference of all microglia for all significant genes between 5xFAD control versus WT control, now with 5xFAD MA10 versus 5xFAD control (*y*‐axis, both amyloid and MA10 infection). Red text demonstrates genes upregulated in the same direction as in the first scatterplot, but downregulated in the MA10 5xFAD versus control 5xFAD comparison. (D) Venn diagram depicting significant up‐ and downregulated genes unique to 5xFAD control versus WT control and 5xFAD MA10 versus 5xFAD control (right, purple), while demonstrating up‐ and downregulated genes commonly shared between the two comparisons. (E) Three‐way Venn diagram of all significantly up‐ and downregulated genes within microglia between the three comparisons: 5xFAD control versus WT control (white), WT MA10 versus WT control (blue), and 5xFAD MA10 versus 5xFAD control (gray). Genes are considered significantly correlated if the log_2_ fold change magnitude is greater than 0.3. (F) Scatterplot of average difference of all astrocytes for all significant genes between 5xFAD control versus WT control and WT MA10 versus WT control. (G) Venn diagram depicting significant up‐ and downregulated genes in astrocytes unique to 5xFAD control versus WT control (left, white) and WT MA10 versus WT control (right, blue), while demonstrating shared dysregulated genes between the two comparisons. (H) Scatterplot of average difference of all astrocytes for all significant genes between 5xFAD control versus WT control. (I)Venn diagram depicting significant up‐ and downregulated genes in astrocytes unique to 5xFAD control versus WT control (left, white) and 5xFAD MA10 versus 5xFAD control (right, gray), while demonstrating shared dysregulated genes between the two comparisons. (J) Three‐way Venn diagram of all significantly up‐ and downregulated genes within astrocytes between the three comparisons: 5xFAD control versus WT control (white), WT MA10 versus WT control (blue), and 5xFAD MA10 versus 5xFAD 48 control (gray).

We have demonstrated that MA10 infection in the periphery shifts the transcriptional state of microglia toward a greater loss of homeostatic markers. To investigate whether these dysregulated responses in microglia are a consequence of peripheral MA10 infection, the presence of Aβ pathology, or unique to the presence of both inflammatory stimuli, we compared the average differences in gene expression within all microglia in our spatial transcriptomic dataset. We plotted all DEGs found in comparing control 5xFAD versus control WT samples (i.e., response to Aβ‐associated pathology) and those found comparing MA10‐infected WT versus control WT (i.e., response to MA10 infection) using the average difference of each gene between the two comparisons (Figure 7A). In response to either Aβ pathology or MA10 infection, microglia shared only four significantly downregulated DEGs (*Csf1r*, *P2ry12*, *Cx3cr1*, *Hexb*), all of which are signature microglial homeostatic markers (Figure 7B). Meanwhile, several genes were uniquely differentially expressed in one inflammatory stimulus over the other. Specific to MA10 infection, microglia demonstrated seven significantly downregulated genes (*Ctsd*, *B2m*, *Ctss*, *C1qb*, *Aldoc*, *Slc1a2*, and *C1qa*). In contrast, microglia demonstrated 60 significantly upregulated genes (e.g., *Apoe*, *Cd74*, *Cst7*, *Ctsd*, *Ctsb*, *Cd9*, *Clec7a*) and 12 significantly downregulated genes (e.g., *Selplg*, *Serinc3*, *Tmem119*, *Hmgb1*, *Camk2a*, *Malat1*, *Tgfbr1*) that were unique to responses to Aβ pathology (Figure 7B). Therefore, while the microglia activation response to peripheral MA10 infection and Aβ pathology downregulated similar homeostatic markers, this comparison highlights the nuanced differences in related groups of genes when considering the magnitude of their inflammatory response.

To investigate the effect of the concurrence of both inflammatory stimuli (e.g., amyloid pathology and MA10 infection) on the gene expression within microglial cells, we plotted all DEGs between control 5xFAD versus control WT (only amyloid pathology) and MA10 5xFAD versus control 5xFAD (both amyloid pathology and MA10 infection) (Figure 7C). Many differential gene expression changes were shared between the two conditions, with several common genes upregulated or downregulated. For example, 17 genes were significantly upregulated within myeloid cells (e.g., *Apoe*, *Iapp*, *Cast*, *Cblb*, *Neu4*, *Spp1*, *Ltbp1*, *Prex1*) in the presence of both inflammatory stimuli together, while five genes were significantly downregulated in both (*Csf1r*, *P2ry12*, *Cx3cr1*, *Tgfbr1*, *Hexb*) (Figure 7D).

To determine which set of DEGs in microglial cells was unique to the interaction of both Aβ pathology and MA10 infection, we compared significant DEGs (*p_adj_* < 0.05) from all microglial cells across the three comparisons for the three conditions: MA10 WT versus control WT (only peripheral MA10 infection), control 5xFAD versus control WT (only Aβ pathology), and MA10 5xFAD versus control 5xFAD (both peripheral MA10 infection and Aβ pathology). As previously demonstrated, this analysis also demonstrated that four homeostatic genes (*Csf1r*, *P2ry12*, *Hexb*, *Cx3cr1*) were significantly downregulated in the context of all three conditions. Among DEGs that were uniquely downregulated only in the context of both amyloid pathology and MA10 infection together were *Cd74*, *Cst7*, *Tyrobp*, *Ctsb*, *H2‐Aa*, *Cd9*, *C1qc*, *Tmsb4x*, and *Cst3*. Meanwhile, these DEGs were actually significantly upregulated in the presence of Aβ pathology or peripheral MA10 infection, independently (Figure 7E). To determine the effects on other cell types within the CNS, we performed a similar analysis with astrocytes (Figure 7F–J), vascular cells, oligodendrocytes, and inhibitory neurons (Figure S8).

Astrocytes showed substantial overlap in transcriptional suppression across conditions. Nine downregulated DEGs were commonly downregulated in response to either Aβ pathology or MA10 infection (*Vegfa*, *Atp1a2*, *Slc6a11*, *Gja1*, *Slc6a11*, *Gpr37l1*, *Plpp3*, *Gstm1*, *Agt*) (Figure 7G). MA10 infection induced 10 additional downregulated DEGs (*Sparcl1*, *Slc4a4*, *Ndrg2*, *Ptprz1*, *Slc1a2*, *Sox9*, *Aldoc*, *Serpine2*, *Cpe*, *Mt3*) and one upregulated DEG (*Mbp*), while Aβ pathology alone resulted in six downregulated (*Hmgb1*, *Malat1*, *Mfge8*, *Bcan*, *Mertk*, *Ptn*) and 10 upregulated DEGs (*Clu*, *Gfap*, *Psen1*, *Tmsb4x*, *Vim*, *Apoe*, *Aqp4*, *Ctsd*, *Dbi*, *Cd81*). Six genes were commonly downregulated with both stimuli (*Atp1a2*, *Mfge8*, *Bcan*, *Gja1*, *Gpr37l1*, *Plpp3*) (Figure 7I), and four genes (*Atp1a2*, *Gpr37l1*, *Gja1*, *Plpp3*) were consistently downregulated across all three conditions (Figure 7J).

Vascular cells shared six downregulated DEGs in response to either Aβ pathology or MA10 infection (*Rgs5*, *Slc1a2*, *Flt1*, *Sparcl1*, *Bsg*, *Pecam1*) (Figure S8B). MA10 infection resulted in six additional downregulated (*Cpe*, *B2m*, *Pltp*, *Sparc*, *Cldn5*, *Vtn*) and four upregulated DEGs (*Slc2a1*, *Vim*, *Apod*, *Acta2*), whereas Aβ pathology alone produced one downregulated (*Slc2a1*) and three upregulated DEGs (*Gfap*, *Psen1*, *Clu*) (Figure S8B). Three genes (*Flt1*, *Bsg*, *Pecam1*) were consistently downregulated under both stimuli and across all three conditions (Figure S8D,E).

Oligodendrocytes showed no shared DEGs between Aβ pathology and MA10 infection (Figure S8G). MA10 infection induced six downregulated (*Pllp*, *Malat1*, *Myrf*, *Mag*, *Ptgds*, *Ugt8a*) and two upregulated DEGs (*Sgk1*, *Mbp*), while Aβ pathology resulted in one downregulated (*Hmgb1*) and 10 upregulated DEGs (*Apoe*, *B2m*, *Clu*, *Cstd*, *Gfap*, *Psen1*, *Pllp*, *Cst7*, *Cd9*, *Plp1*) (Figure S8G). No shared DEGs were detected under combined or all three conditions (Figure S8J).

Inhibitory neurons shared three downregulated DEGs in response to either Aβ pathology or MA10 infection (*Atp2a2*, *Hspa8*, *Sst*) (Figure S8L). MA10 infection induced seven additional downregulated (*Hsp90aa1*, *Tcf7l2*, *Gnas*, *Sparcl1*, *Ywhag*, *Cpe*, *Sparc*) and four upregulated DEGs (*Ppp1r1b*, *Penk*, *Arpp21*, *Pcp4*), whereas Aβ pathology resulted in five downregulated (*Cplx1*, *Syp*, *Meg3*, *Snhg11*, *Penk*) and one upregulated DEG (*Psen1*) (Figure S8L). With both stimuli, two DEGs were commonly downregulated (*Penk*, *Sst*) (Figure S8N), and only *Sst* remained downregulated across all three conditions (Figure S8O).

## 4 DISCUSSION

Recent studies have reported that infection with either SARS‐CoV‐2 or mouse‐adapted SARS‐CoV‐2 (MA10) has led to the presence of gliosis and other CNS changes in animal models in the absence of virus in the brain, arguing that peripheral inflammatory responses to viral infection can impact CNS resident cell response.^23^, ^24^, ^25^ While COVID‐19 most commonly presents with respiratory symptoms, neurological symptoms have been extensively documented and can emerge during acute infection and persist beyond viral clearance.^49^ The full extent of neuropathological changes in the CNS that result from SARS‐CoV‐2 infection is still not fully understood. Given the association between COVID‐19, neuroinflammation, and neurodegenerative processes, further characterization of CNS alterations following infection and their potential contribution to neurodegeneration is needed.

To address these questions, we infected aged (10 to 14 months old) C57BL/6 WT and 5xFAD mice with mouse‐adapted SARS‐CoV‐2 (MA10) to assess neuropathological changes following chronic infection and examine infection‐induced alterations in brain gene expression. We utilized aged 5xFAD mice to evaluate the effects of SARS‐CoV‐2 infection on a brain with already established amyloid pathology. This approach was intended to model the clinical scenario of infection in elderly patients with pre‐existing dementia. It remains possible that infection at a younger age, prior to the onset of significant Aβ deposition, might more profoundly influence the trajectory or magnitude of subsequent pathology – a question that warrants further investigation.

As both microglia and astrocytes can contribute to neuroinflammatory and neurodegenerative processes,^18^ changes in microglia and astrocyte number and volume were quantified. In contrast to prior reports suggesting infection‐induced microgliosis,^24^ we observed no significant changes in microglial number or volume in the brains of infected WT or 5xFAD mice compared to uninfected controls. Similarly, plasma levels of interleukin‐1β (IL‐1β), a cytokine previously implicated in neuroinflammation, plasticity, and memory deficits,^50^ were unchanged following infection.

Additionally, we detected no increase in MAC2+ monocytes or macrophages within the brains of MA10‐infected WT or 5xFAD mice at day 21 p.i. Astrocyte number and volume were also unchanged at day 21 p.i., consistent with reports showing minimal astrocytic changes at later time points following infection^51^ and despite transient increases in GFAP expression reported during the acute infection.^23^ Uninfected 5xFAD mice exhibited increased astrocyte number and volume in the cortex relative to WT mice, consistent with astrocyte activation in response to progressive Aβ plaque accumulation.^29^ The absence of overt microglial or astrocytic activation following MA10 infection may reflect the timing of analysis, the age of the mice, the use of a mouse‐adapted viral strain, or the infectious dose employed.

While our histological analysis did not reveal overt changes in glial numbers or volume, we acknowledge the limitations of immunohistochemistry in fully capturing complex activation phenotypes. Future studies utilizing flow cytometry would be beneficial to provide a more robust, quantitative assessment of protein‐level changes in microglial homeostatic markers. Furthermore, while MAC2 staining suggested minimal peripheral myeloid cell infiltration, more comprehensive profiling is needed to rule out the involvement of other peripheral immune populations, such as T cells, which could subtly influence the CNS transcriptomic environment.

Both MA10‐infected WT and 5xFAD mice exhibited changes in gene expression that were associated with neuronal dysfunction. RNASeq analysis indicates an upregulation in genes associated with reduced LTP in WT mice, and downregulation in genes associated with synaptic function and plasticity in 5xFAD mice. Given the established role of LTP as a cellular correlate of learning and memory and its impairment in memory disorders,^52^ these transcriptional changes likely contribute to cognitive deficits reported in both clinical and experimental studies. Notably, 5xFAD mice exhibited a greater number of DEGs compared to WT mice at day 21 p.i., likely reflecting heightened vulnerability associated with pre‐existing Aβ pathology.

Although RNA‐seq analyses suggested neuronal and synaptic dysfunction, MA10 infection did not induce or exacerbate overt neuropathological hallmarks of AD. Specifically, we observed no changes in dystrophic neurites, Aβ plaque burden in 5xFAD mice, or neuronal loss or synapse loss in either genotype. These findings were somewhat unexpected given reports that coronavirus proteins, including the envelope (E) protein, possess amyloidogenic properties^16^ and that MA10 infection can influence other AD‐related pathologies, such as phosphorylated tau.^24^, ^25^ Together, these data indicate that, at the viral dose employed, MA10 infection does not drive overt neurodegeneration or exacerbate classical AD pathology.

Despite the absence of overt neuropathological changes, widespread transcriptomic dysregulation revealed subtle but potentially consequential molecular alterations across multiple CNS‐resident cell types. Significant changes in homeostatic gene expression were observed in microglia, astrocytes, oligodendrocytes, inhibitory neurons, and vascular endothelial cells, suggesting early signatures of neuroinflammation and homeostatic disruption that may precede longer‐term neurodegenerative processes.

In both WT and 5xFAD mice, microglia exhibited downregulation of established homeostatic genes, including *Hexb*, *Csf1r*, *P2ry12*, and *Cx3cr1*, genes typically suppressed during microglial activation or transition to a disease‐associated microglia (DAM) phenotype.^34^ Downregulation of these genes suggests a shift away from a homeostatic microglial state, consistent with observations in viral encephalitis and neurodegenerative disorders, such as AD.^53^ Notably, in MA10‐infected 5xFAD mice only, microglia showed upregulation of genes involved in immune regulation (*Cblb*),^54^ protease inhibition (*Cast*),^55^ lysosomal degradation (*Neu4*),^56^ and inflammatory signaling and metabolic dysfunction (*Iapp*).^57^ Increased expression of *Apoe*, a defining feature of DAMs, supports the emergence of a reactive microglial phenotype.^58^

Astrocytes showed downregulation of key genes involved in neurotransmitter regulation (*Slc1a2*), gap junctional communication (*Gja1*), phospholipid metabolism (*Plpp3*), ion homeostasis (*Atp1a2*), and neurodevelopment (*Ptprz1*). Reduced expression of *Slc1a2*, encoding the astrocytic glutamate transporter EAAT2/GLT‐1, is associated with excitotoxicity and neuronal injury in aging and neurodegenerative disease.^59^ Downregulation of *Atp1a2* may impair potassium clearance and neuronal excitability,​^60^​ while reduced *Gja1* (connexin43) expression disrupts astrocyte communication and has been associated with neuroinflammation.^61^ *Plpp3* downregulation disrupts lipid‐mediated signaling, impairing astrocyte–neuron communication, leading to deficits in neurotransmission.^62^ *Ptprz1* has established supporting roles enabling neurogenesis and plasticity.^63^ Together, these changes indicate compromised astrocytic support functions that may contribute to neuronal dysfunction without causing overt degeneration.

In addition to astrocytes and microglia, oligodendrocytes, inhibitory interneurons, and vascular endothelial cells also displayed pronounced transcriptional changes. Oligodendrocytes showed downregulation of myelination‐associated genes, including *Mag*,^64^ *Ugt8a*,^65^ and *Pllp*,^66^ as well as *Malat1*, which has neuroprotective functions.^67^ Upregulation of *Sgk1* has been associated with metabolic stress in the brain, neurodegeneration and cognitive impairment, and a potential mediator of tau phosphorylation.^68^ These changes suggest oligodendrocyte dysfunction or early demyelinating processes, potentially contributing to not only neurodegenerative processes but also white matter abnormalities reported in post‐COVID imaging studies.^69^

Inhibitory interneurons showed the reduction in expression of *Ndrg4*, *Sst*, *Npy*. *Sst* (somatostatin), and *Npy* (neuropeptide Y), which are critical neuropeptides involved in regulating neuronal excitability and synaptic transmission.^42^ Their downregulation may indicate impaired inhibitory control, potentially contributing to disrupted cortical circuitry and excitatory‐inhibitory imbalance. Similarly, the reduction in *Ndrg4*, a gene involved in synaptic vesicle trafficking and neuronal survival.^43^ Overall findings suggest the presence of early synaptic vulnerability in the post‐infection brain.

Endothelial cells displayed downregulation of *Bsg*, *Cldn5*, and *Pecam1*, all of which are vital for BBB integrity.^70^, ^71^, ^72^ *Cldn5* encodes a tight junction protein critical for BBB permeability, and its downregulation may reflect early BBB disruption, a finding consistent with neuropathological reports in COVID‐19 patients.^22^ Conversely, upregulation of *Apod* and *Acta2* may indicate vascular remodeling, suggesting vascular changes in the brain not related to BBB disruption and dysfunction but associated with remodeling of affected vessels, in a maladaptive manner, due to the stress caused by an initial insult, such as inflammation and infection.^73^, ^74^

Results from this study contribute to growing information providing insight into mechanisms by which SARS‐CoV‐2 infection contributes to neurologic deficits. Our results have shown there are dramatic changes in genes impacting homeostatic gene functions in microglia, astrocytes, and oligodendroglia, as well as neurons and vascular endothelial cells. These subtle, yet potentially important, changes in cell function can occur in the absence of viral infection of the CNS and impact neurologic functions, including behavior and memory. Furthermore, MA10 infection did not impact the severity of AD‐associated neuropathology in the 5xFAD model, although there were numerous changes in glial and neuron gene expression within the brains. We believe that one mechanism by which this may occur is in response to the peripheral inflammation that occurs in response to MA10 infection of the lungs leading to increased proinflammatory cytokines/chemokines that can access the CNS and alter resident cell function. Collectively, these findings underscore the need for further research to elucidate the molecular mechanisms underlying COVID‐19‐related CNS changes and their long‐term impact on neurodegenerative processes.

## 5 CONCLUSION

Ongoing studies have demonstrated that SARS2 infection impacts the severity of clinical disease in elderly patients with established dementia. This study was undertaken to better understand the effects of SARS2 infection on impacting the severity of AD pathology using experimental infection of aged 5xFAD and WT mice with MA10, a mouse‐adapted SARS2. While MA10 infection of 5xFAD mice did not result in either increased neuroinflammation or a reduction in Aβ plaque size/volume, there was a dramatic effect on the expression of genes encoding homeostatic function in glia, neurons, and vascular endothelial cells in both 5xFAD and WT mice. These findings argue that in the absence of viral infection of the brain, peripheral MA10 infection and the ensuing inflammatory response result in subtle yet sustained gene expression within the brain, which has implications for long‐term neurologic deficits.

## AUTHOR CONTRIBUTIONS

S.F., K.N.G., and T.E.L. designed the study. R.S.B. provided mouse‐adapted SARS‐CoV‐2 and assisted in experimental design. S.F., L.Z., G.M.O., Y.C., C.P., and K.F. performed experiments and analyzed data. G.P.S., L.Z., and D.A.N. performed and analyzed cytokine/Luminex data. R.A.E. performed histologic analysis of lungs. S.F. and D.I.J. performed bulk RNA‐seq analysis. K.I.T. performed spatial transcriptomics. K.I.T., L.Z., D.I.J., and L.L. analyzed spatial transcriptomic data. S.F., L.Z., D.I.J., and T.E.L. wrote the manuscript.

## CONFLICT OF INTEREST STATEMENT

The authors declare no conflicts of interest. Author disclosures are available in the Supporting Information.

## CONSENT STATEMENT

No human subjects were used for the present study. Therefore, consent was not necessary.

## Supporting information



Supporting Information

Supporting Information

Supporting Information

Supporting Information

Supporting Information

Supporting Information

Supporting Information

Supporting Information

Supporting Information

## Data Availability

Protocols, data, and results will be available via the AD Knowledge Portal.
