# Supplementary material for: Infection of 5xFAD mice with a mouse‐adapted SARS‐CoV‐2 does not alter Alzheimer's disease neuropathology yet induces widespread changes in gene expression across diverse cell types
Source: Alzheimers Dement. 2026 Apr 24;22(4):e71394. doi: 10.1002/alz.71394 (PMC13108251; doi:10.1002/alz.71394)
Supplement: Supplementary file 2 — Supporting Information [file ALZ-22-e71394-s008.pdf]

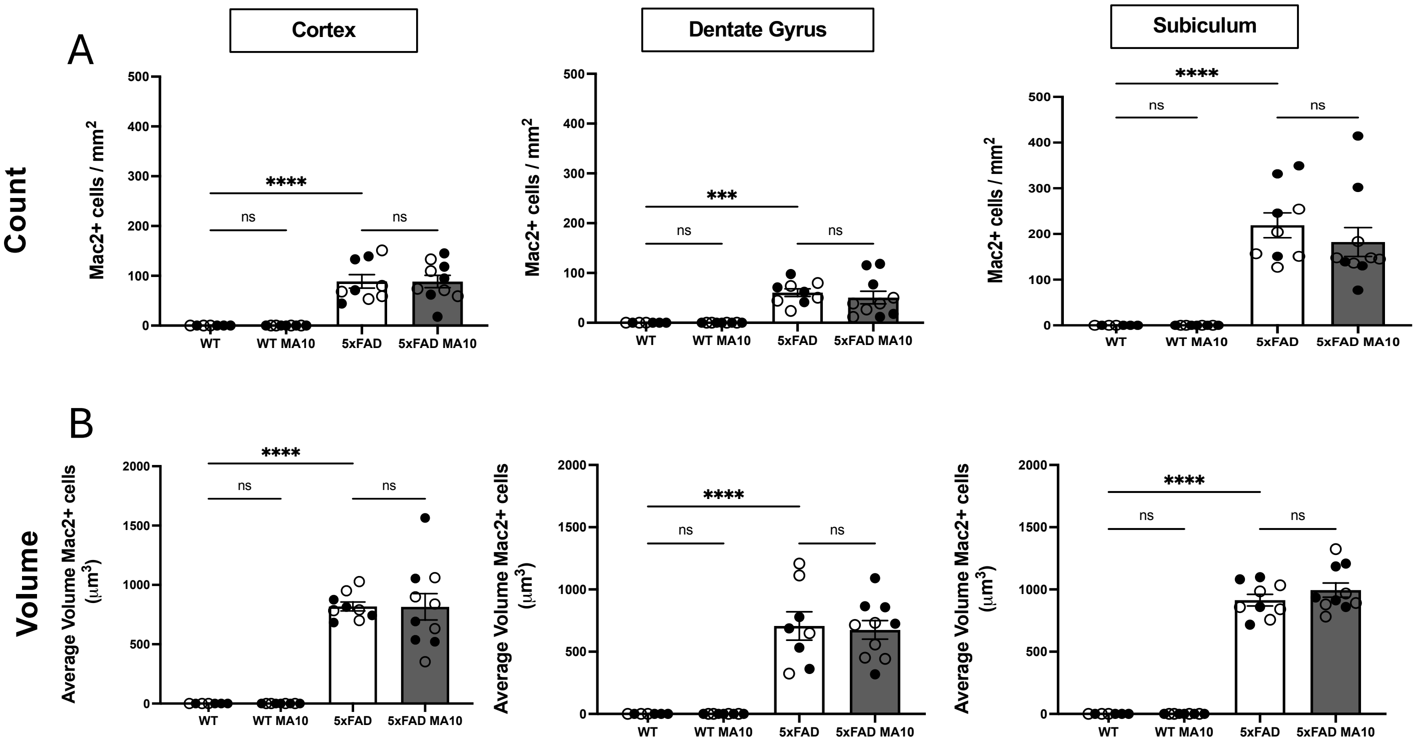

Supplemental Figure 1. (**A**) Quantification of Mac2$^+$ cells and their volumes (**B**) in the cortex, dentate gyrus, and subiculum of MA10-infected WT and 5xFAD mice at day 21 p.i. Immunohistological data were analyzed using two-way ANOVA. Tukey's post-hoc test was employed to examine biologically relevant interactions. Female and male mice are indicated by open or closed circles respectively. Data are represented as mean ±SEM; *** $p \leq 0.001$, **** $p \leq 0.0001$.
