# Supplementary material for: Infection of 5xFAD mice with a mouse‐adapted SARS‐CoV‐2 does not alter Alzheimer's disease neuropathology yet induces widespread changes in gene expression across diverse cell types
Source: Alzheimers Dement. 2026 Apr 24;22(4):e71394. doi: 10.1002/alz.71394 (PMC13108251; doi:10.1002/alz.71394)
Supplement: Supplementary file 3 — Supporting Information [file ALZ-22-e71394-s002.pdf]

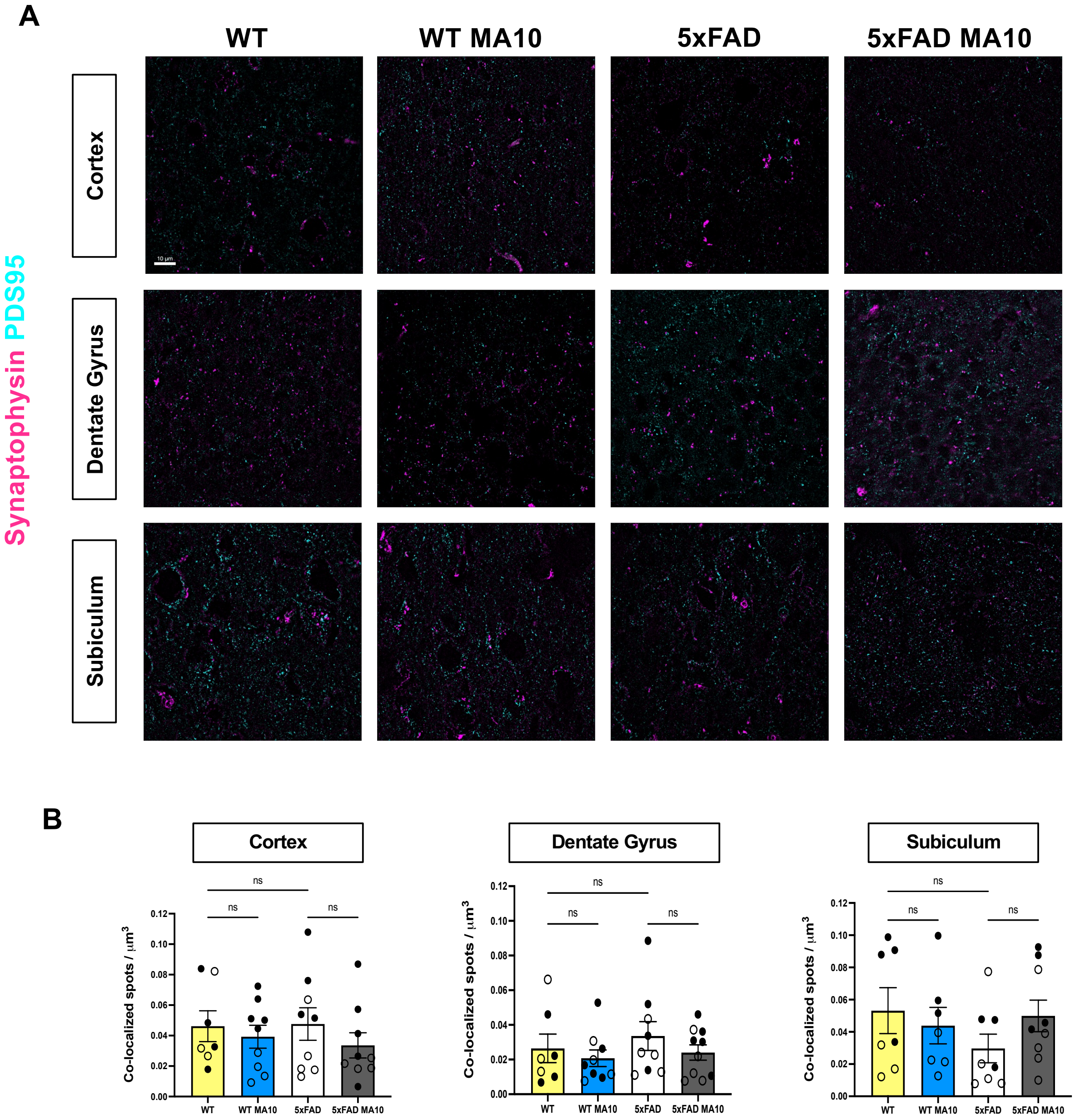

Supplemental Figure 2. MA10 infection and synaptic vesicle density in WT and 5xFAD mice. Brains of MA10-infected uninfected WT and 5xFAD mice at day 21 p.i. were immunostained with synaptophysin for presynaptic elements (magenta) and PSD-95 for postsynaptic elements (turquoise). (**A**) Representative super-resolution images at 40X objective of cortex, dentate gyrus, and subiculum from infected and uninfected mice are shown. (**B**) Quantification of synaptophysin+ and PSD-95+ colocalized spots per µm3. Immunohistological data were analyzed using two-way ANOVA. Tukey's post-hoc test was employed to examine biologically relevant interactions. Female and male mice are indicated by open or closed circles respectively. Data are represented as mean ±SEM. Scale bar in (A) = 10 µm.
