# Supplementary material for: Infection of 5xFAD mice with a mouse‐adapted SARS‐CoV‐2 does not alter Alzheimer's disease neuropathology yet induces widespread changes in gene expression across diverse cell types
Source: Alzheimers Dement. 2026 Apr 24;22(4):e71394. doi: 10.1002/alz.71394 (PMC13108251; doi:10.1002/alz.71394)
Supplement: Supplementary file 4 — Supporting Information [file ALZ-22-e71394-s004.pdf]

Cell segmentation examples

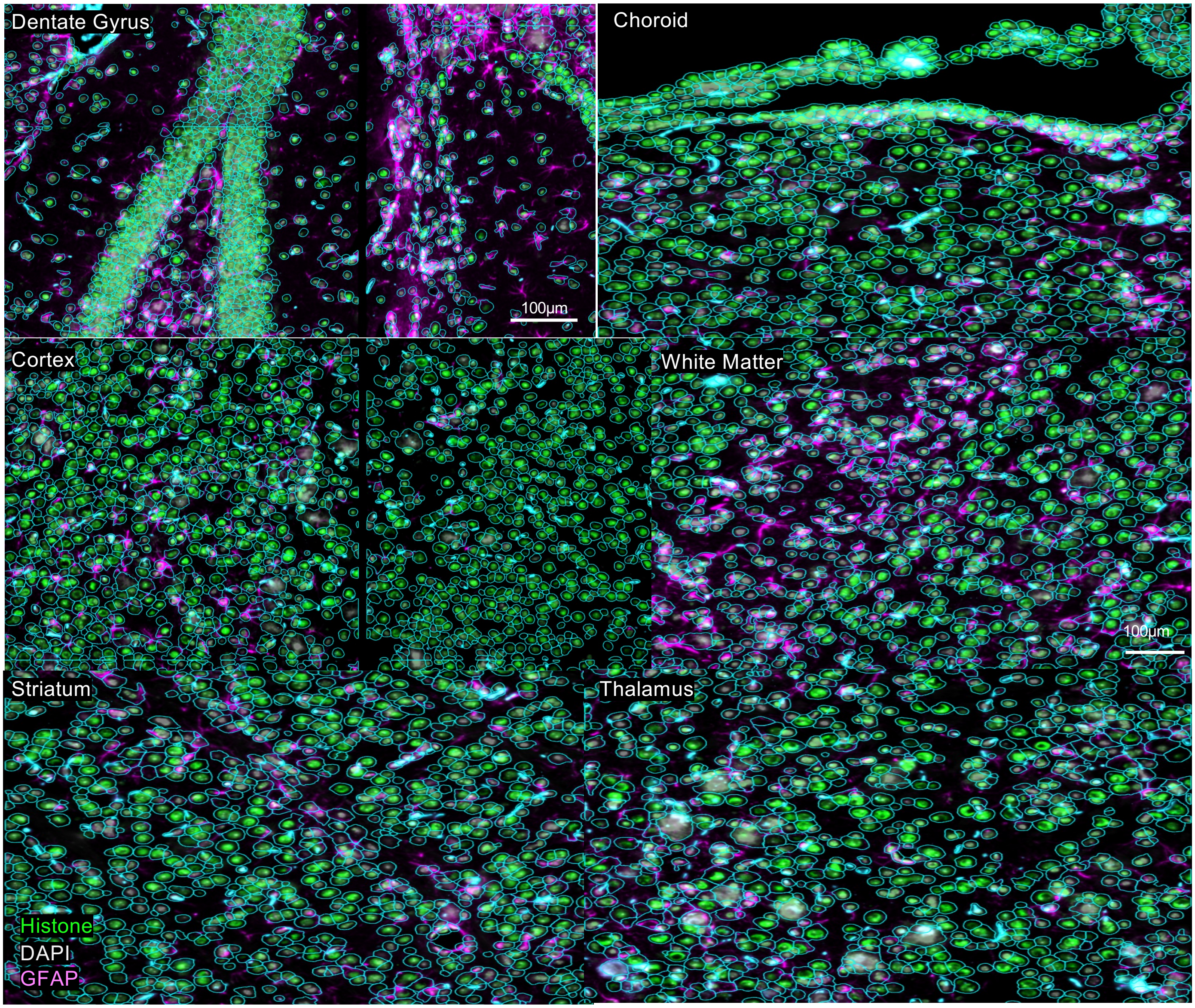

Supplemental Figure 3. Representative images demonstrating cell segmentation in dentate gyrus, choroid plexus, cortex, white matter, striatum and thalamus. Cells were imaged with rRNA (not shown), histone, DAPI, and GFAP markers and segmented automatically.
