# Supplementary material for: Infection of 5xFAD mice with a mouse‐adapted SARS‐CoV‐2 does not alter Alzheimer's disease neuropathology yet induces widespread changes in gene expression across diverse cell types
Source: Alzheimers Dement. 2026 Apr 24;22(4):e71394. doi: 10.1002/alz.71394 (PMC13108251; doi:10.1002/alz.71394)
Supplement: Supplementary file 5 — Supporting Information [file ALZ-22-e71394-s003.pdf]

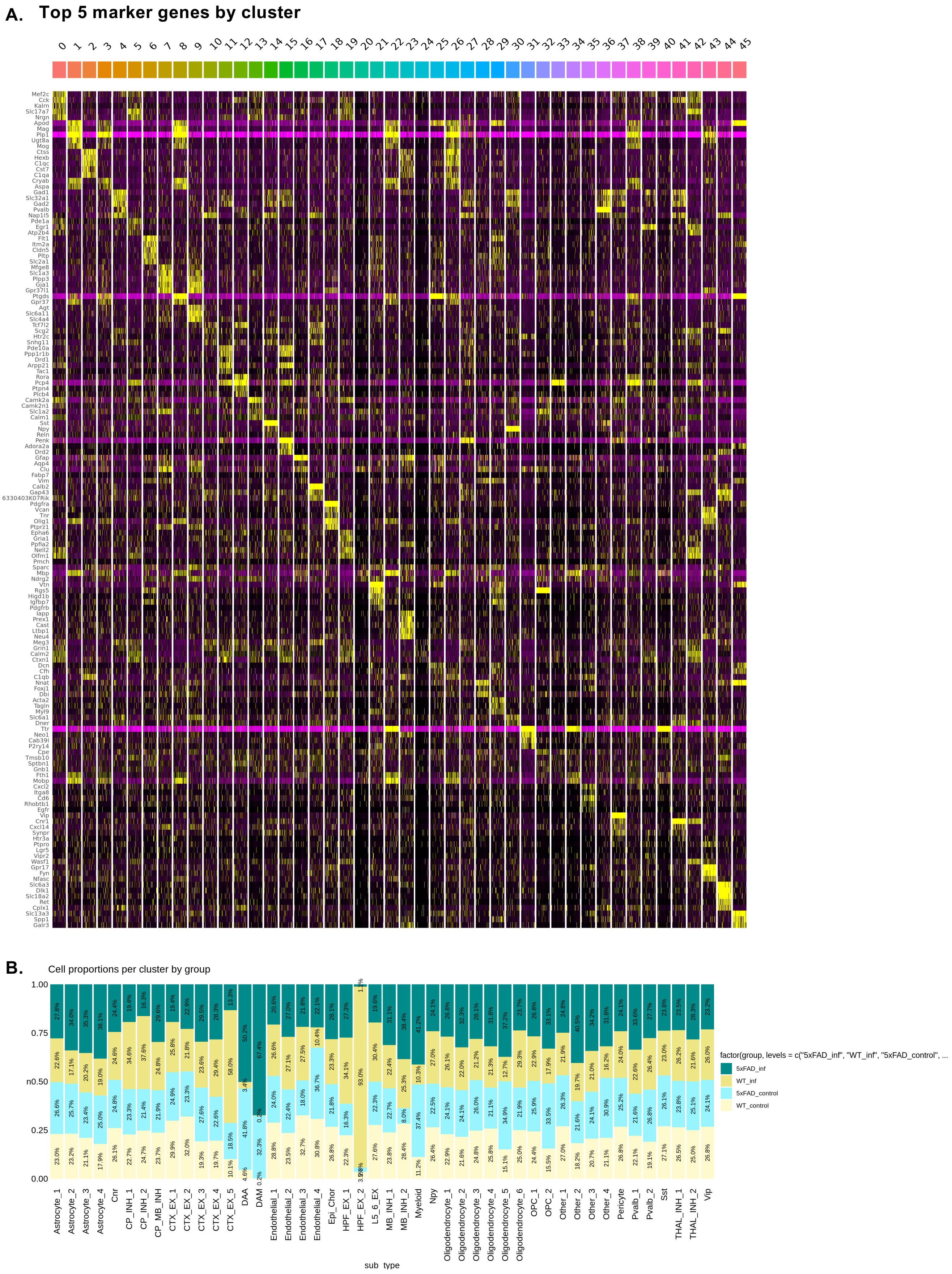

Supplemental Figure 4. (**A**) Heatmap of top 5 marker genes for all spatial transcriptomics subclusters. (**B**) Proportional distribution of Seurat clusters across experimental groups. Bar plots display relative abundance of each cluster in each group.
