# Supplementary material for: Infection of 5xFAD mice with a mouse‐adapted SARS‐CoV‐2 does not alter Alzheimer's disease neuropathology yet induces widespread changes in gene expression across diverse cell types
Source: Alzheimers Dement. 2026 Apr 24;22(4):e71394. doi: 10.1002/alz.71394 (PMC13108251; doi:10.1002/alz.71394)
Supplement: Supplementary file 6 — Supporting Information [file ALZ-22-e71394-s001.pdf]

Cell clusters in XY space, all brains

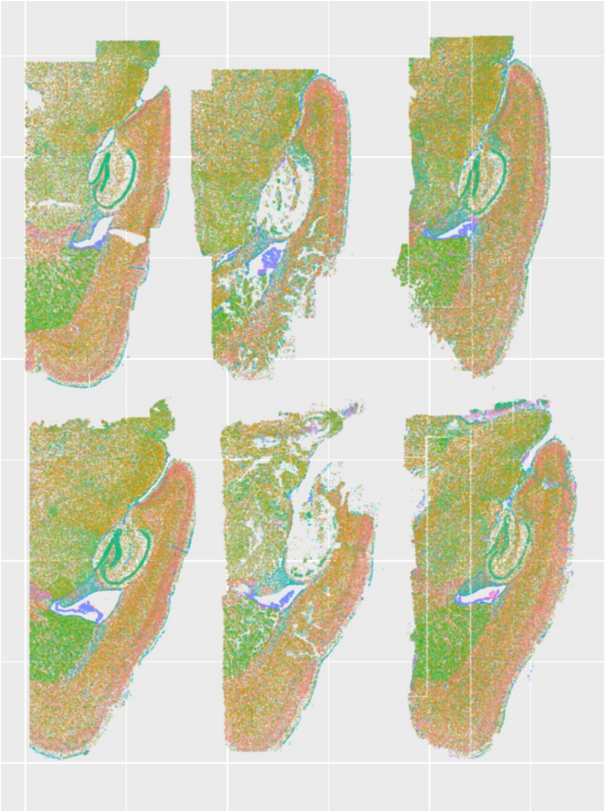

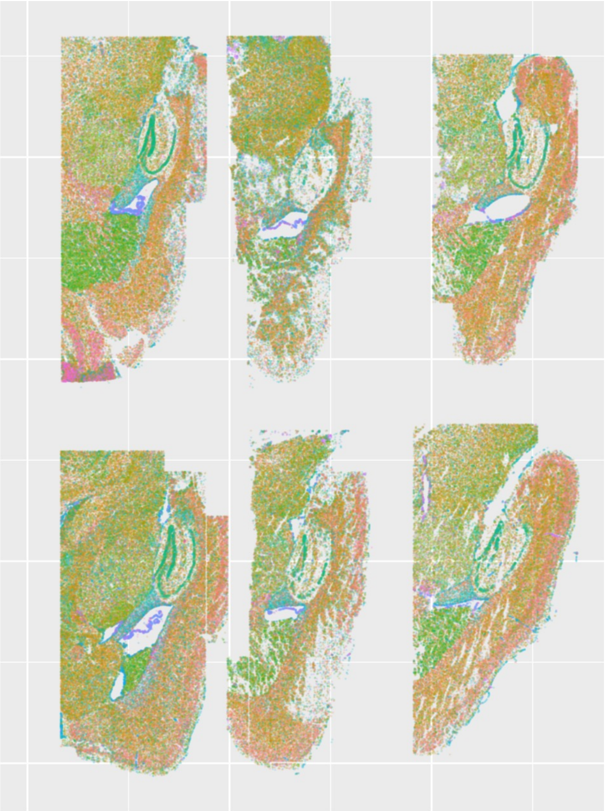

Cluster	Cell type	Sub-type
**0**	Exitatory_Neuron	CTX_EX_1
**1**	Olygodendrocyte	Olygodendrocyte_1
**2**	Myeloid	Myeloid
**3**	Olygodendrocyte	Olygodendrocyte_2
**4**	Inhibitory_Neuron	Pvalb_1
**5**	Exitatory_Neuron	L5_6_EX
**6**	Vascular	Endothelial_1
**7**	Astrocyte	Astrocyte_1
**8**	Olygodendrocyte	Olygodendrocyte_3
**9**	Astrocyte	Astrocyte_2
**10**	Inhibitory_Neuron	MB_INH_1
**11**	Inhibitory_Neuron	CP_INH_1
**12**	Inhibitory_Neuron	THAL_INH_1
**13**	Exitatory_Neuron	CTX_EX_2
**14**	Inhibitory_Neuron	Sst
**15**	Inhibitory_Neuron	CP_INH_2
**16**	Astrocyte	DAA
**17**	Inhibitory_Neuron	THAL_INH_2
**18**	OPC	OPC_1
**19**	Exitatory_Neuron	HPF_EX_1
**20**	Astrocyte	Astrocyte_3
**21**	Vascular	Pericyte
**22**	Olygodendrocyte	Olygodendrocyte_4
**23**	Myeloid	DAM
**24**	Exitatory_Neuron	CTX_EX_3
**25**	Other	Other_1
**26**	Olygodendrocyte	Olygodendrocyte_5
**27**	Inhibitory_Neuron	CP_MB_INH
**28**	Astrocyte	Astrocyte_4
**29**	Vascular	Endothelial_2
**30**	Inhibitory_Neuron	Npy
**31**	Epithelial	Epi_Chor
**32**	Vascular	Endothelial_3
**33**	Exitatory_Neuron	CTX_EX_4
**34**	Other	Other_2
**35**	Vascular	Endothelial_4
**36**	Inhibitory_Neuron	Pvalb_2
**37**	Inhibitory_Neuron	Vip
**38**	Olygodendrocyte	Olygodendrocyte_6
**39**	Exitatory_Neuron	CTX_EX_5
**40**	Other	Other_3
**41**	Inhibitory_Neuron	Cnr
**42**	Exitatory_Neuron	HPF_EX_2
**43**	OPC	OPC_2
**44**	Inhibitory_Neuron	MB_INH_2
**45**	Other	Other_4

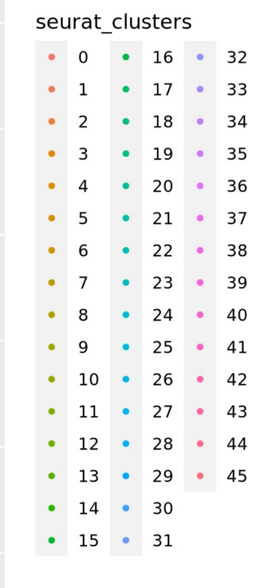

Supplemental Figure 5. 46 clusters plotted in XY space in all 6 brains from WT (n=3) and 5xFAD (n=3) mice, both infected and uninfected, at day 21 p.i. (n=3/group).
