# Supplementary material for: Infection of 5xFAD mice with a mouse‐adapted SARS‐CoV‐2 does not alter Alzheimer's disease neuropathology yet induces widespread changes in gene expression across diverse cell types
Source: Alzheimers Dement. 2026 Apr 24;22(4):e71394. doi: 10.1002/alz.71394 (PMC13108251; doi:10.1002/alz.71394)
Supplement: Supplementary file 7 — Supporting Information [file ALZ-22-e71394-s006.pdf]

Infected WT vs. con WT

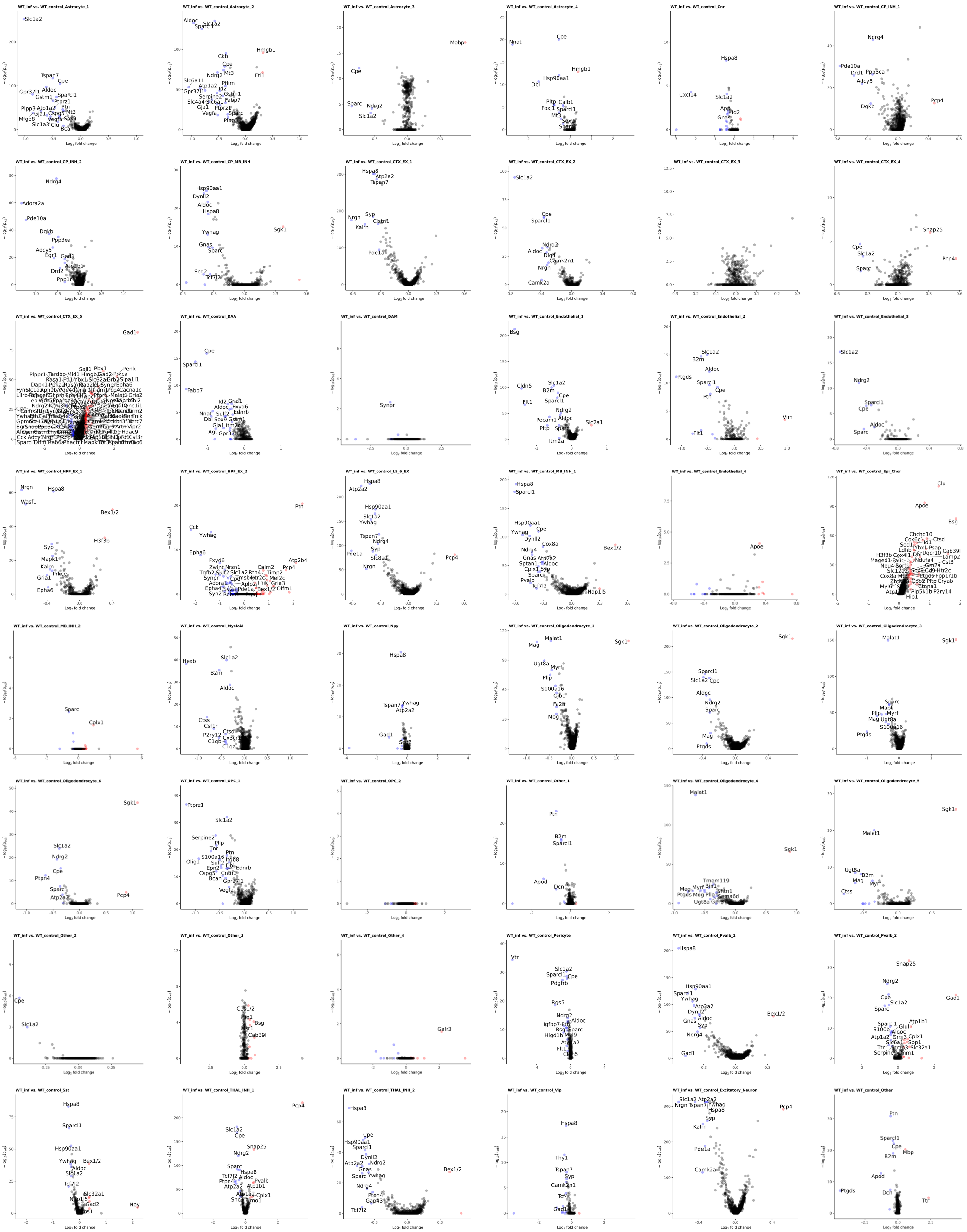

Supplemental Figure 6. Volcano plots for all spatial transcriptomics subcluster in MA10 infected WT vs Control WT.
