# Supplementary material for: Infection of 5xFAD mice with a mouse‐adapted SARS‐CoV‐2 does not alter Alzheimer's disease neuropathology yet induces widespread changes in gene expression across diverse cell types
Source: Alzheimers Dement. 2026 Apr 24;22(4):e71394. doi: 10.1002/alz.71394 (PMC13108251; doi:10.1002/alz.71394)
Supplement: Supplementary file 8 — Supporting Information [file ALZ-22-e71394-s007.pdf]

Infected 5xFAD vs. con 5xFAD

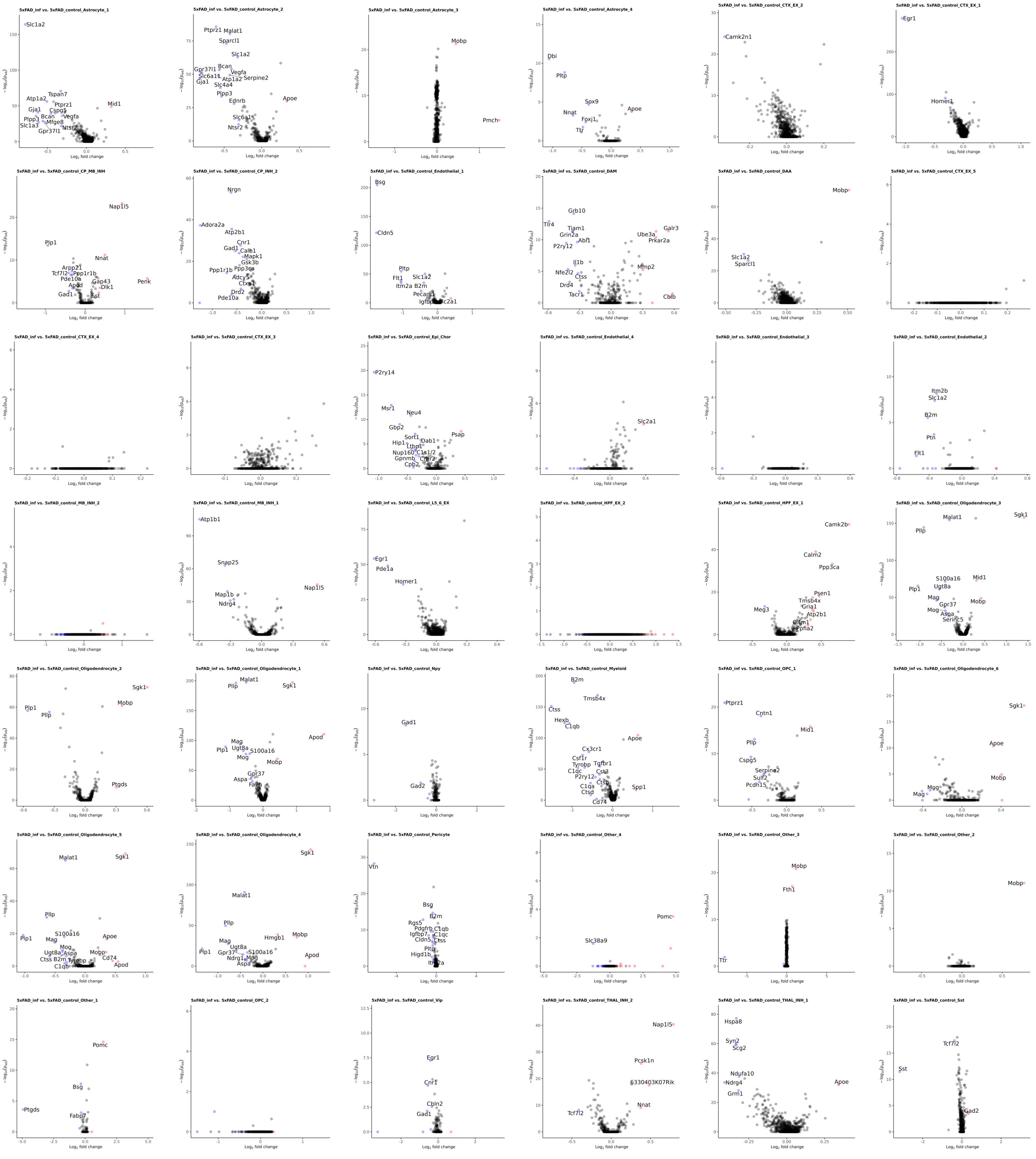

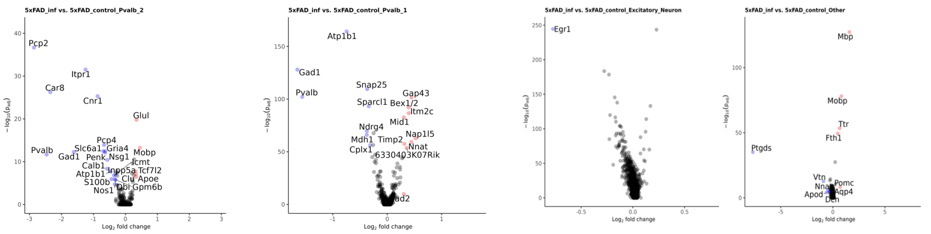

Supplemental Figure 7. Volcano plots for all spatial transcriptomics subcluster in MA10 infected 5xFAD vs Control 5xFAD.
