# Supplementary material for: Infection of 5xFAD mice with a mouse‐adapted SARS‐CoV‐2 does not alter Alzheimer's disease neuropathology yet induces widespread changes in gene expression across diverse cell types
Source: Alzheimers Dement. 2026 Apr 24;22(4):e71394. doi: 10.1002/alz.71394 (PMC13108251; doi:10.1002/alz.71394)
Supplement: Supplementary file 9 — Supporting Information [file ALZ-22-e71394-s009.pdf]

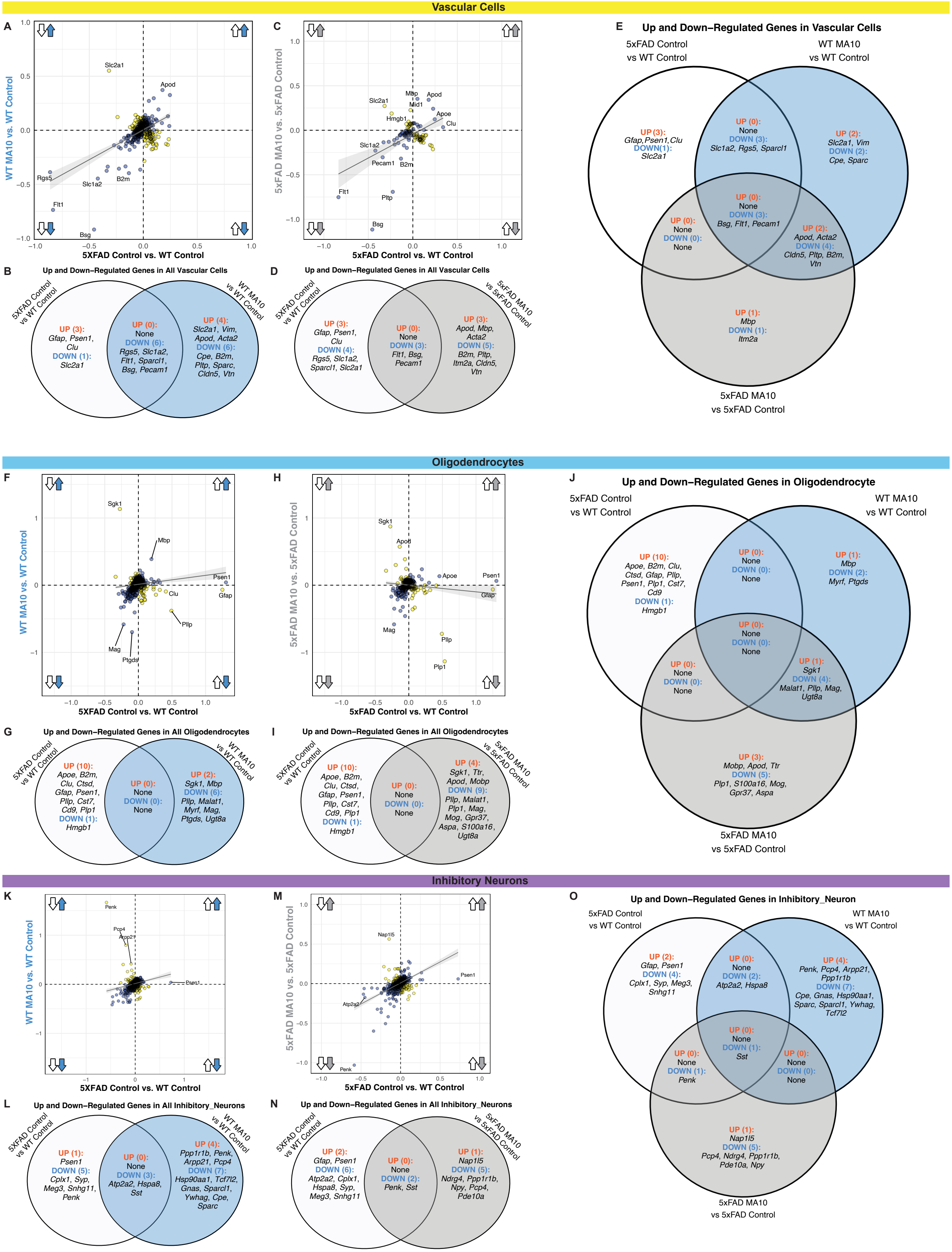

Vascular Cells
A
WT MA10 vs. WT Control
5XFAD Control vs. WT Control
Slc2a1
Apod
Rgs5
Slc1a2
B2m
Flt1
Bsg
B
Up and Down–Regulated Genes in All Vascular Cells
5XFAD Control vs WT Control
WT MA10 vs WT Control
UP (3): Gfap, Psen1, Clu DOWN (1): Slc2a1
UP (0): None DOWN (6): Rgs5, Slc1a2, Flt1, Sparcl1, Bsg, Pecam1
UP (4): Slc2a1, Vim, Apod, Acta2 DOWN (6): Cpe, B2m, Pltp, Sparc, Cldn5, Vtn
C
5XFAD MA10 vs. 5xFAD Control
5XFAD Control vs. WT Control
Mbp
Apod
Mid1
Slc2a1
Hmgb1
Apoe
Clu
Slc1a2
Pecam1
B2m
Flt1
Pltp
Bsg
D
Up and Down–Regulated Genes in All Vascular Cells
5XFAD Control vs WT Control
5xFAD MA10 vs 5xFAD Control
UP (3): Gfap, Psen1, Clu DOWN (4): Rgs5, Slc1a2, Sparcl1, Slc2a1
UP (0): None DOWN (3): Flt1, Bsg, Pecam1
UP (3): Apod, Mbp, Acta2 DOWN (5): B2m, Pltp, Itm2a, Cldn5, Vtn
E
Up and Down–Regulated Genes in Vascular Cells
5xFAD Control vs WT Control
WT MA10 vs WT Control
UP (3): Gfap,Psen1,Clu DOWN(1): Slc2a1
UP (0): None DOWN (3): Slc1a2, Rgs5, Sparcl1
UP (2): Slc2a1, Vim DOWN (2): Cpe, Sparc
UP (0): None DOWN (3): Bsg, Flt1, Pecam1
UP (0): None DOWN (0): None
UP (2): Apod, Acta2 DOWN (4): Cldn5, Pltp, B2m, Vtn
UP (1): Mbp DOWN (1): Itm2a
5xFAD MA10 vs 5xFAD Control
Oligodendrocytes
F
WT MA10 vs. WT Control
5XFAD Control vs. WT Control
Sgk1
Mbp
Psen1
Gfap
Clu
Pllp
Mag
Ptgds
G
Up and Down–Regulated Genes in All Oligodendrocytes
5XFAD Control vs WT Control
WT MA10 vs WT Control
UP (10): Apoe, B2m, Clu, Ctsd, Gfap, Psen1, Pllp, Cst7, Cd9, Plp1 DOWN (1): Hmgb1
UP (0): None DOWN (0): None
UP (2): Sgk1, Mbp DOWN (6): Pllp, Malat1, Myrf, Mag, Ptgds, Ugt8a
H
5XFAD MA10 vs. 5xFAD Control
5XFAD Control vs. WT Control
Sgk1
Apod
Apoe
Psen1
Gfap
Mag
Pllp
Plp1
I
Up and Down–Regulated Genes in All Oligodendrocytes
5XFAD Control vs WT Control
5xFAD MA10 vs 5xFAD Control
UP (10): Apoe, B2m, Clu, Ctsd, Gfap, Psen1, Pllp, Cst7, Cd9, Plp1 DOWN (1): Hmgb1
UP (0): None DOWN (0): None
UP (4): Sgk1, Ttr, Apod, Mobp DOWN (9): Pllp, Malat1, Plp1, Mag, Mog, Gpr37, Aspa, S100a16, Ugt8a
J
Up and Down–Regulated Genes in Oligodendrocyte
5xFAD Control vs WT Control
WT MA10 vs WT Control
UP (10): Apoe, B2m, Clu, Ctsd, Gfap, Pllp, Psen1, Plp1, Cst7, Cd9 DOWN (1): Hmgb1
UP (0): None DOWN (0): None
UP (1): Mbp DOWN (2): Myrf, Ptgds
UP (0): None DOWN (0): None
UP (0): None DOWN (0): None
UP (1): Sgk1 DOWN (4): Malat1, Pllp, Mag, Ugt8a
UP (3): Mobp, Apod, Ttr DOWN (5): Plp1, S100a16, Mog, Gpr37, Aspa
5xFAD MA10 vs 5xFAD Control
Inhibitory Neurons
K
WT MA10 vs. WT Control
5XFAD Control vs. WT Control
Penk
Pcp4
Arpp21
Psen1
M
5XFAD MA10 vs. 5xFAD Control
5XFAD Control vs. WT Control
Nap1l5
Psen1
Atp2a2
Penk
O
Up and Down–Regulated Genes in Inhibitory_Neuron
5xFAD Control vs WT Control
WT MA10 vs WT Control
UP (2): Gfap, Psen1 DOWN (4): Cplx1, Syp, Meg3, Snhg11
UP (0): None DOWN (2): Atp2a2, Hspa8
UP (4): Penk, Pcp4, Arpp21, Ppp1r1b DOWN (7): Cpe, Gnas, Hsp90aa1, Sparc, Sparcl1, Ywhag, Tcf7l2
UP (0): None DOWN (1): Sst
UP (0): None DOWN (1): Penk
UP (0): None DOWN (0): None
UP (1): Nap1l5 DOWN (5): Pcp4, Ndrg4, Ppp1r1b, Pde10a, Npy
5xFAD MA10 vs 5xFAD Control
L
Up and Down–Regulated Genes in All Inhibitory_Neurons
5XFAD Control vs WT Control
WT MA10 vs WT Control
UP (1): Psen1 DOWN (5): Cplx1, Syp, Meg3, Snhg11, Penk
UP (0): None DOWN (3): Atp2a2, Hspa8, Sst
UP (4): Ppp1r1b, Penk, Arpp21, Pcp4 DOWN (7): Hsp90aa1, Tcf7l2, Gnas, Sparcl1, Ywhag, Cpe, Sparc
N
Up and Down–Regulated Genes in All Inhibitory_Neurons
5XFAD Control vs WT Control
5xFAD MA10 vs 5xFAD Control
UP (2): Gfap, Psen1 DOWN (6): Atp2a2, Cplx1, Hspa8, Syp, Meg3, Snhg11
UP (0): None DOWN (2): Penk, Sst
UP (1): Nap1l5 DOWN (5): Ndrg4, Ppp1r1b, Npy, Pcp4, Pde10a

Supplemental Figure 8. Investigating gene dysregulation within vascular cells, oligodendrocytes and inhibitory neurons in the presence of amyloid pathology, peripheral MA10 infection, or both concurrently. (**A**) Scatterplot of the average difference of all vascular cells for all significant genes between 5xFAD Control vs. WT Control and WT MA10 vs. WT Control. (**B**) Venn diagram depicting the significant up-and down-regulated genes in vascular cells unique to 5xFAD Control vs. WT Control (left, white) and WT MA10 vs. WT Control (right, blue), while demonstrating shared dysregulated genes between the two comparisons. (**C**) Scatterplot of the average difference of all vascular cells for all significant genes between 5xFAD Control vs. WT Control. (**D**) Venn diagram depicting the significant up-and down-regulated genes in vascular cells unique to 5xFAD Control vs. WT Control (left, white) and 5xFAD MA10 vs. 5xFAD Control (right, grey), while demonstrating shared dysregulated genes between the two comparisons. (**E**) Three-way Venn diagram of all significantly up- and down-regulated genes within vascular cells between the three comparisons: 5xFAD Control vs. WT Control (white), WT MA10 vs. WT Control (blue), and 5xFAD MA10 vs. 5xFAD Control (grey). (**F**) Scatterplot of the average difference of all oligodendrocytes for all 50 significant genes (padj < 0.05) between 5xFAD Control vs. WT Control (x-axis, only amyloid pathology) and WT MA10 vs. Control WT (y-axis, only MA10 infection) comparisons. Arrows indicate direction of dysregulation for each comparison. Directly correlated genes (blue) occur in the same direction for both comparisons (i.e., both up-regulated or both down-regulated), while inversely correlated genes (orange) occur in opposite directions for each comparison. Linear regression line demonstrates the relationship between the two comparisons. (**G**) Venn diagram depicting the significant up- and down-regulated genes unique to the 5xFAD Control vs. WT Control (left, white) and WT MA10 vs. WT Control (right, blue), while demonstrating up- and down-regulated genes commonly shared across the two comparisons (middle). (**H**) Scatterplot of the average difference of all oligodendrocytes for all significant genes between 5xFAD Control vs. WT Control, now with 5xFAD MA10 vs. 5xFAD Control (y-axis, both amyloid and MA10 infection). Red text demonstrates genes up-regulated in the same direction in the first scatterplot, but down-regulated in the MA10 5xFAD vs. Control 5xFAD comparison. (**I**) Venn diagram depicting the significant up- and down-regulated genes unique to 5xFAD Control vs. WT Control and 5xFAD MA10 vs. 5xFAD Control (right, purple), while demonstrating up- and down-regulated genes commonly shared between the two comparisons. (**J**) Three-way Venn diagram of all significantly up- and down-regulated genes within oligodendrocytes between the three comparisons: 5xFAD Control vs. WT Control (white), WT MA10 vs. WT Control (blue), and 5xFAD MA10 vs. 5xFAD Control (grey). Genes are considered significantly correlated if the log2 Fold Change magnitude is greater than 0.3.(**K**) Scatterplot of the average difference of all inhibitory neurons for all significant genes between 5xFAD Control vs. WT Control and WT MA10 vs. WT Control. (**L**) Venn diagram depicting the significant up- and down- regulated genes in inhibitory neurons unique to 5xFAD Control vs. WT Control (left, white) and WT MA10 vs. WT Control (right, blue), while demonstrating shared dysregulated genes between the two comparisons. (**M**) Scatterplot of the average difference of all inhibitory neurons for all significant genes between 5xFAD Control vs. WT Control. (**N**) Venn diagram depicting the significant up- and down-regulated genes in inhibitory neurons unique to 5xFAD Control vs. WT Control (left, white) and 5xFAD MA10 vs. 5xFAD Control (right, grey), while demonstrating shared dysregulated genes between the two comparisons. (**O**) Three-way Venn diagram of all significantly up- and down-regulated genes within inhibitory neurons between the three comparisons: 5xFAD Control vs. WT Control (white), WT MA10 vs. WT Control (blue), and 5xFAD MA10 vs. 5xFAD Control (grey).
